# Reading English-Language Haiku: Processes of Meaning Construction Revealed by Eye Movements

**DOI:** 10.16910/jemr.10.1.4

**Published:** 2017-02-28

**Authors:** Müller Hermann J., Geyer Thomas, Günther Franziska, Kacian Jim, Pierides Stella

**Affiliations:** General and Experimental Psychology, LMU, Munich Germany; Department of English and American Studies, LMU, Munich Germany; The Haiku Foundation, Winchester, VA, USA

**Keywords:** poetry reading, English-language haiku, eyetracking, gaze, attention, neuro-cognitive poetics

## Abstract

In the present study, poets and cognitive scientists came together to investigate the construction of meaning in the process of reading normative, 3-line English-language haiku (ELH), as found in leading ELH journals. The particular haiku which we presented to our readers consisted of two semantically separable parts, or images, that were set in a 'tense' relationship by the poet. In our sample of poems, the division, or cut, between the two parts was positioned either after line 1 or after line 2; and the images related to each other in terms of either a context-action association (context-action haiku) or a conceptually more abstract association (juxtaposition haiku). From a constructivist perspective, understanding such haiku would require the reader to integrate these parts into a coherent 'meaning Gestalt', mentally (re-)creating the pattern intended by the poet (or one from within the poem's meaning potential). To examine this process, we recorded readers' eye movements, and we obtained measures of memory for the read poems as well as subjective ratings of comprehension difficulty and understanding achieved. The results indicate that processes of meaning construction are reflected in patterns of eye movements during reading (1st-pass) and re-reading (2nd- and 3rd-pass). From those, the position of the cut (after line 1 vs. after line 2) and, to some extent, the type of haiku (context-action vs. juxtaposition) can be 'recovered'. Moreover, post-reading, readers tended to explicitly recognize a particular haiku they had read if they had been able to understand the poem, pointing to a role of actually resolving the haiku's meaning (rather than just attempting to resolve it) for memory consolidation and subsequent retrieval. Taken together, these first findings are promising, suggesting that haiku can be a paradigmatic material for studying meaning construction during poetry reading.

To see a World in a Grain of Sand

And a Heaven in a Wild Flower

William Blake, Auguries of Innocence (ll. 1–2)

## Introduction

We all experience a sense of unity and wholeness, simple as well as revelatory, in moments of insight, such as when a wildflower opens up to us with all its completeness and beauty. Writers and poets attempt to share this experience by recreating it in the mind of the reader. How this may be achieved, what processes of (re)construction and insight go on in the reader’s ‘mindbrain’, is a question that has concerned poets for a long time. More recently, it has also come into the focus of scientists in the areas of *cognitive*, and *neuro-cognitive*, *poetics* (henceforth referred to as *neuro-/cognitive poetics*).

The present study – a co-operation between (haiku) poets and cognitive scientists (psychologists, linguists) – positions itself within this broader, interdisciplinary field.

Specifically, the aim of neuro-/cognitive poetics is to understand the mental ‘processing’ of literary texts (reception, comprehension, appreciation, emotional response), including poetry, using the concepts and methodological approaches of neuro-/cognitive psychology. When applied to well-constrained literary material, these methods allow reliable and valid inferences to be drawn about the underlying neuro-/cognitive mechanisms.

To meet this requirement, Kliegl (personal communication, 2010) advocates the use of short forms of poetry (micropoetry). Here, we take this recommendation further by arguing that *English-language haiku* (henceforth abbreviated as ELH) provide a paradigmatic form of poetry for this purpose (note that *haiku* is both the singular term for one haiku poem and the plural form for multiple haiku). Although haiku poems vary widely, in their normative (three- or one-line, variable line-length) format, they share properties that make them eminently suitable for understanding how the mind-brain makes sense and meaning of literary texts. One key feature of several techniques and devices used by haiku poets in three-line haiku is placing two images in relation to – or juxtaposed with – one another, often in surprising ways, across what is referred to as a *cut* or *caesura*, inviting the reader to construct, or contribute to the construction of, the haiku’s meaning (see, e.g. 49 , and below).

On this background, the present study was designed to investigate the reading of ELH using eye-movement recording, combined with (cognitive) measures of memory for the read material as well as subjective ratings of comprehension difficulty and of the understanding achieved. Although (the patterns of) eye movements during reading and memory measures obtained postreading are purely behavioral data, they permit inferences to be drawn about some of the underlying neuro/cognitive processes involved in the construction of meaning. The succession of eye fixations within a piece of text tells us where the reader’s attention is allocated to: from where information (from the visual word-encoding stage to semantic processing levels) is extracted over time and integrated in the representation of global meaning ( 25, 3 ). And memory measures can tell us something about the *depth of processing*( 18, 70 ) engaged in.

In order to set the stage for the present study, there follows (i) a brief introduction to the field of neuro/cognitivepoetics, elaborating some key distinctions, followed by (ii) arguments in favor of using haiku as study material. The latter section includes a brief exposition of the literary form of ELH and reasons why this form is particularly suitable for investigating processes of meaning construction in the reading of poetic texts. Subsequently, (iii) the concrete questions addressed in the present study are developed, along with an exposition of the design and methodology employed.

### Neuro-cognitive Poetics

While we understand relatively little, as yet, about what happens in the mind-brain when people read literary texts ( 29, 73 ), studying the processing of literary language – in particular, poetry – has been recognized as “well suited to compactly demonstrate the complexities with which our brains construct the world in and around us”, permitting processes of “thought, language, … and images” (cognition) to be brought together with those of “play, pleasure, and emotion” (motivation/emotion) ( 45 , p. 2). Accordingly, attempts to bridge the gap between literature/literary studies and neuro-science have recently become more frequent, giving rise to the field of neuro/cognitive poetics.

Jacobs and colleagues synthesized this growing body of work into a (qualitative) model of literary reading (for an overview, see 45 ) : an attempt “to make explicit … a number of hypotheses about mental processes theoretically involved in (written) literature reception and their interrelations at the three main levels of inquiry …[:] the neuronal, subjective-experiential, and objectivebehavioral” ( 45 , p. 14). Drawing on the cognitive-poetics literature (e.g., 95 ) , the model assumes that all literary texts, including even single words in isolation, consist of, and transport, *background* [BG] and *foreground* [FG] *features*, in various mixture ratios.

The BG–FG distinction can be traced back to *Gestalt* theory (e.g., 107, 108 ) : the notion that (e.g., visual) perception involves lawful processes of organization that integrate basic perceptual elements (e.g., visual features such as lines, curves, color patches, etc.) into coherent wholes, or *Gestalten* (‘figures’). The wholes thus created are perceptually foregrounded, in the focus of attention (whereas the ungrouped elements remain in the amorphous background), and have a meaning of their own which is *other* than the sum of their parts (and, in fact, alters the interpretation of the elements).

These fundamental notions from, originally, perception theory were later extended to other psychological fields, including problem solving (conceived as a process of mental reorganization; e.g., 22, 60 ) , and to other domains, including the study of language: *cognitive linguistics*(e.g., 67, 68, 98 ; see also 20, 102 for an overview) and, importantly, *cognitive poetics*(e.g., 95 ). The central idea is that, since complex processes of mental organization are invoked in the *ception*( 97, 98 ) of literary texts, literary construction and appreciation encourages play with perceptions, conceptions, and expectations, as well as shifts in the relationship between background and foreground. Stockwell concludes that: “Figure and ground are therefore the basic features of literary stylistic analysis” ( 95 , p. 15).

BG features are the elements of a text that evoke a feeling of familiarity in the reader: familiar words, phrases, and images; on the level of knowledge structures: familiar situation models, socio-cultural codes, and affective scripts. As such, BG features “facilitate immersive processing … through the automatic (implicit) activation of familiar cognitive schemata, situation models, and affective responses” ( 95 , p. 16). Lines and sections of text containing predominantly BG elements are interpretationally shallow, and the reading act is “little disturbed by attention-capturing features and the higher cognitive processes … [of] mental situation-model and eventstructure building (59, … 93)” ( 95 , p. 16). This gives rise to a feeling of immersion: “the reader is absorbed by and transported into the text world, being in a ‘flow’ … (43)” ( 95 ,p. 16).

In this fluent/linear reading mode, which is characterized by larger eye movements and shorter fixations, the fundamental processes of reading – word recognition and eye guidance – are predominantly controlled by the reading networks of the left brain hemisphere ( 91 ). And immersive processes are supported by the ancient (mammalian) affective core systems described by Panksepp ( 77, 78 ).

Technically, BG features are being used by the (literary) author “to evoke the underlying associative network indirectly in the [reader’s] mind … to control the stream of thought” – in James’s ( 47 ) terms, to control the relationship between the current focal *nucleus* of the stream and other, potential thoughts and feelings forming the *fringe*, that is, “how internal processes in the reader’s mindbrain fill-in gaps in the text … through associations that form the basis of memories, imagination, and anticipations” ( 45 , p. 7).

FG features of a text, by contrast, relate more directly to elements in the focus of attention. Importantly, FG features, such as unusual form elements (including, in poetry, the use of line breaks) and semantic ambiguities, may be brought in a relationship of tension or conflict with the BG elements, interrupting the flow by capturing attention. In such situations, the repertory of standard cognitive and affective schemata no longer suffices to make meaning, “defamiliaris[ing] what the reader thought s/he recognized, leading to a distrust of the expectations aroused and a reconsideration of seemingly straightforward discrepancies that are unwilling to accommodate themselves to these patterns” (Iser, as cited in 45 , p. 7). This induces a disfluent/non-linear – potentially poetic/aesthetic – reading mode, characterized by “evaluative [(self-)reflective] processing, … not only (automatically) recognizing words, but ‘seeing’, ‘hearing’, or ‘smelling’ them. Eye movement behavior slows down, as do thoughts and feelings: they expand …” ( 45 , p. 16). This serves the effortful process of closing *meaning Gestalts*( 43 ), that is, discovering or constructing new meanings from the multitude of meaning potentials that the (skillfully crafted) text affords – involving the adaptation of schemata and situation models and processes of integration and synthesis.

Reaching the end of this *aesthetic trajectory* ( 30 ) is rewarding: “after initial moments of familiar recognition, followed by surprise, ambiguity, and tension, the closure of meaning gestalts [releases the tension and is] … occasionally supplemented by an ‘aha’ experience … or feeling of good fit, ‘rightness’, or harmony …” ( 45 , p. 16).

This mode of reading is characterized by smaller eye movements and longer fixations, and associated with increased activity in the left-hemispheric dorsolateral reading circuit (e.g., in left inferior frontal gyrus), the ancient lust, play, and seek (affective) system ( 77, 78 ), and, importantly, with significantly increased activity in the right hemisphere’s associative networks. Furthermore, greater employment of FG features (i.e., abstractness/ defamiliarization) in poetic texts correlates with higher ratings of aesthetic emotions/beauty ( 71 ), and spontaneous (implicit) processes of aesthetic evaluation engender activations in brain regions associated with reward/pleasure and beauty ( 104, 65 ). This is consistent with the long recognition, in literary theory, of “[t]he rewarding character of novelty and FG through artful deviation” ( 45 , p. 11): according to Berlyne ( 4 ), incongruity or deviation can produce a pleasurable degree of arousal (one of the two variables determining affective reactions), and according to Iser ( 43 ), closing an open meaning Gestalt is associated with pleasure.

### Haiku as paradigmatic study material

In the neuro-/cognitive poetics literature, various types of stimulus material have been used to examine what happens in the mind-brain when people read literary texts, ranging from extended prose texts (e.g., sections from Harry Potter novels; 42 ) to, usually longer forms of, poetry (e.g., 114 ). These developments have been supported by methodological advances of formally analyzing and characterizing larger (sections of) texts (e.g., in terms of processing fluency or emotion potential; 42 ), providing larger-scale descriptors whose mental correlates can be examined by using neuro-/cognitive methodology, such as fMRI. However, despite such advances, these methods (still) require relatively well-constrained stimulus material to be optimally applicable, in order to support reliable and valid inferences about the underlying neuro/cognitive mechanisms.

One important criterion in this regard is repeatability of measurement: a pre-condition for discerning stable patterns, across texts and participants, that can be theoretically interpreted as reflecting well-defined mental processes. Arguably, texts at the micro-level pole of written material fulfill this criterion more readily than larger sections of texts, or entire stories or novels (at the other pole). Given this, short forms of poetry may provide particularly suitable material for studying the reading of poetic texts. This approach has been advocated by Kliegl (personal communication, 2010), who used a short story, attributed to Hemingway, comprising only six words: “For sale: baby shoes, never worn” to illustrate this point. He notes that most readers resonate with the deep sadness of this story, and goes on to state: “Our experiments test whether such contrasts in subjective experience [as evoked in reading Hemingway’s short story] lead to detectable bodily responses [as reflected, e.g., in eye movements]; they do not *reduce* the experience to the bodily responses or their symbolic representations.”

Taking this further, we propose that the specific form of ELH, and its characteristic features of juxtaposition, cut, its syntactic, temporal, and dynamic aspects, and its use of keywords and imagery/nouns, fulfills two desiderata: different individual haiku are (i) compositionally well constrained and similar in structure, while varying in meaning/content, thus allowing for systematic variation and repeated measurement; (ii) haiku engage a rich set of mental functions with the minimum of linguistic means (using everyday, unadorned language, devoid of stylistic poetic devices), thus offering a potent literary form for investigating processes of meaning construction, including closure: the resolution of surprise induced by the juxtaposed images. As illustrated in the next section, ELH contain an interesting mixture of, and interplay between, background and foreground features, providing a paradigmatic study material for neuro-/cognitive poetics.

**Figure 1. Stimulus material.**
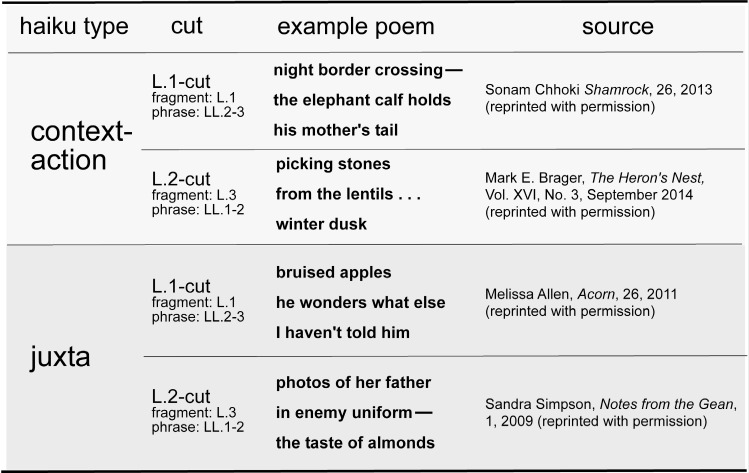
Example haiku from the sample used in the study, for each of the four haiku type x cut position conditions. As an illustration of the interplay between the BG-FG modes of processing in haiku reading, take, for example, S. Chhoki's poem: the fragment “night border crossing” in this haiku will invoke, in the reader's mind, a grounding context/situation model, setting up expectations as to the range of possibilities to follow depending on personal, cultural, and/or other associations, most likely involving humans/the narrator crossing a border clandestinely, invoking feelings of danger, worry, suspense. The subsequent phrase (following an explicit cut marker) “the elephant calf / holds his mother's tail” will challenge this situation model, jolting the reader into foreground mode. In this mode, the reader can adapt/change the model from ‘human’ to ‘animal’ agents, though effecting this adaptation/change is compounded by the realization that animals don’t know anything about human-defined borders. The final line then adds an element (that is shared by humans) of touch/touching/feeling of security/containment, as well as resolution, which is put against the suspense set up in the first line.

Originating in Japan, haiku developed its own identity in the English-speaking West as English-language haiku(ELH)(e.g., 53 ). See Figure 1 for examples. A brief poem, unrhymed, normative haiku unfolds over three lines, in a short–long–short line pattern, with, as a rule, fewer than 17 syllables in total, not necessarily arranged in the earlier 5–7–5 syllable pattern. (Note that there are variants to this arrangement, such as shape poems, poems of varying lines, and free-form haiku; here, we focus on the three-line norm, permitting comparison with other variants – in particular, the less frequent, but equally normative one-line haiku, known as *monoku* – in future work.) Furthermore, haiku records a moment of insight into the nature of the world, in an effort to share it with others (e.g., 49 ). The contemporary haiku poet aims to convey her/his experience of that moment in the present (including recollected as well as imagined moments) in words that render it so concisely and directly – without commenting, explaining, or marveling at the experience – and, at the same time, so suggestively – making the words expand in the reader’s mind into a multitude of images and feelings – that it is possible for the reader to re-create and share that moment and the insight it encapsulates. (Interestingly, this directly links haiku with scientific notions of *‘embodied cognition’*; e.g., 3 ).

This process is aided by the facts (i) thathaiku use ordinary, everyday vocabulary, images, and concepts, importantly including season keywords/phrases (such as *cherry blossom*, *harvest moon*, *snow*, or *new year’s eve*) that refer to a season, occasion, or aspect of the environment, and (ii) that haiku have a rich, and long, tradition known to, and shared by, the poets and their (initiated) readership. While keywords such as *harvest moon* may not be entirely transparent to the uninitiated, 21st-century reader, everyone would develop a fitting set of associations to *new year’s eve*. Such keywords thus evoke in the reader’s mind, ‘in a nutshell’, a season of the year and associations, literary connections, and scripts that ground the poem. That is, they provide background(BG) features that allow for an element of immersion on the part of the reader.

In addition, the development of haiku is skillfully crafted by the poet, using the stylistic devices of formal, foregrounding (FG) elements of pacing (for an illustration, see, e.g., the commentary by Jason Charnesky on John Martone’s haiku “forest skull’s”; 13 ) and line breaks (the latter at least in traditional, three-line haiku) as well as introducing the element of cut, that is, a break point or gap between two (at first glance) often seemingly disparate parts or images. This is what constitutes the poetic device of *juxtaposition*: two images (1) – or, in Reichhold’s terms ( 86 ), *fragment* and *phrase* parts – are juxtaposed side by side in a more or less tense relationship, inviting comparison of the haiku’s constituent elements – inviting the reader to unravel the significance of the moment the poet presents; to reconstruct the experience and/or construct his/her own meaning.

Note that the strength of the juxtaposition varies between different types of haiku, such as between *haiku of juxtaposition* and *context–action haiku*. In context–action haiku, “one of the images … establishes the setting where the haiku moment is experienced; the other suggests the activity which caught the notice of the poet’s imagination” ( 49 ) – so, for the reader, the gap between the two images is more straightforward to close (an example, by J. Kacian, 48 , would be: “drowned moth— / the wax hardens / around it”). In juxtaposition haiku, by contrast, “two images not obviously related by context or action are paired” (an example, by M. Allen 1 , would be: “bruised apples / he wonders what else / I haven’t told him”) – with a clear, recognizable pause, break, or gap between the two disparate parts. [Apart from syntax, ELH often use punctuation to indicate and emphasize the cut, though the cut itself would normally be clearly discernible even without such markers ( 36 ).] This gives rise to a startling, defamiliarizing, almost uncanny experience and acts as an invitation to processes of reflection and reappraisal (this is one sense in which haiku may be distinguished from other forms of micropoetry and microtexts). As Paul W. MacNeil 72 put it: “… it is in the space between [the parts], that space created by the break or cut, that haiku are found.”

Thus, juxtapositions (especially those in juxtaposition haiku) give rise, at first, to feelings of *discrepancy* and *surprise*, activating the play-and-seek system and recruiting mental problem-solving processes to reduce the surprise and release the tension (consistent with Friston's 32 fundamental free-energy principle of brain function). Resolution of the ‘puzzle’, filling-in of the gap, realization of how the juxtaposed images go together, achieving integration/coherence and closure of the meaning Gestalt – depending on the reader’s psychological, cultural, and/or educational constitution – gives rise to what is referred to as *haiku moment*, which may involve an ‘aha’ experience, aesthetic appreciation, and feelings of reward. This potential has been described as “haiku’s mysterious power to cause in the reader’s consciousness a sudden shift, literally a new way of seeing” ( 17 ). Note, in this context, that haiku is a form of poetry that is interpretationally open, providing ample space for the reader to contribute: the meaning Gestalt ultimately formed by the reader may diverge more or less strongly from that intended by the author.

Given this aesthetic trajectory, we propose that haiku provide an ideal study medium for neuro-/cognitive poetics: the constructive device of juxtaposition, within the context of the brevity and compositional consistency of the form, makes haiku highly attractive for the scientific investigation of central processes that go on in the reader’s mind-brain while reading and appreciating poetic texts.

### Aim and Rationale of the Present Study

The present study positions itself within the larger context of a project investigating the reading, reception, and appreciation of haiku in a more comprehensive manner, using a combination of neuro-/cognitive methods (see also 35, 81 ). The current study used eye-movement recording, combined with post-reading memory and subjective rating measures, to explore how readers of normative ELH scan the poem to derive sense and meaning. – Note that there is a rich literature on what eye-movement measures can reveal about processes of reading (for reviews, see 25, 89 see also supporting material S1 for a brief introdcution).

ELH is written in a variety of approaches ( 7 ) and formats (e.g., from the standard three-line haiku to four-, two-, and one-line haiku). Here, we focus on the normative three-line haiku, with a cut either at the end of line 1 (L.1-cut, i.e., the fragment part is in line 1) or at the end of line 2 (L.2-cut, i.e., the fragment is in line 3). Also, although various schemes have been suggested to classify haiku, here we look into two types: context–action haiku and juxtaposition haiku (see 49 , and above).

Briefly, in context–action haiku, one component (image) of the haiku, the *fragment*, provides the context (e.g., *fragment*: “night border crossing–”) and the other, the *phrase*, describes an action set within this context (*phrase*: “the elephant calf holds / his mother’s tail”; ( 15 ). Both images, although each relatively familiar, are set in a relationship with one another by the poet. In juxtaposition haiku, by contrast, there is no straightforward (familiar) context–action relationship, that is, the images juxtaposed are more jarring, in a relationship of tension that needs to be resolved (e.g., “photos of her father / in enemy uniform– / the taste of almonds”; 92 ). The cut, which is further emphasized by the “–” mark in the examples, is orthogonal to the type of haiku, that is: independently of the type (context–action vs. juxtaposition), the cut can occur after line 1 or after line 2 (in the examples, line breaks are indicated by slashes). See Figure 1 above for further examples of context–action and juxtaposition haiku, and L.1-cut and L.2-cut haiku.

Given these distinctions, the primary aim of the present, exploratory study was to examine the patterns of eye movements during haiku reading, with participants being instructed to try to achieve an understanding of the haiku they were presented with. The study’s central questions were as follows: (how) do the eye-movement patterns reflect (i) the fact *that* there is a cut and (ii) *where* in the text the cut is positioned? And (iii) (how) may cut effects be modulated by the type of haiku? Taking a reader/recipient-centered approach on the reading and processing of texts in general (see below; see also 16 , pp. 31–33; 103, 58 ), and of haiku as poetic texts in particular (see, e.g. 52 ), we aimed at gaining first insights into the effects of the poetic form characteristic of haiku on the readers’ processes of text analysis and meaning construction.

Note that at this stage of the project, and given the ‘state of the art’ in (empirical) neuro-/ cognitive poetics research, it is hard to formulate a solid theoretical grounding, based on general reading research, for the more intricate, moment-to-moment processes going on in the reading of haiku (see also 106 ). Accordingly, we take a more ‘bottom-up’, exploratory approach, by asking whether cut-position effects (and their modulation by haiku type) would *at all* be reflected (or be discernible *at all*) in the eye-movement patterns. To our knowledge, there are no reports in the (eye-movement) literature of cut-like effects in the reading of poetry, while we are aware of (unsuccessful) attempts to establish such effects (e.g., in the reading of sonnets; see Discussion for a more detailed consideration of these attempts). Thus, arguably, only if such effects are actually demonstrable in the reading and re-reading patterns do we have an *empirical* handle that puts us in a position to ask more complex questions about the on-line processes of meaning construction and resolution. To establish this using haiku (rather than longer forms of poetry) as reading material is the primary aim of the present study.

Given this, we nevertheless formulated a few general (and seemingly ‘common-sense’) predictions about the cut effects: the position of the cut in the haiku was expected to have a major influence on the scanning pattern, with the fragment line (i.e., line 1 in L.1-cut haiku and, respectively, line 3 in L.2-cut haiku) – as the line requiring (semantic/conceptual) integration (as well as, in context–action haiku, providing the scene setting) – receiving the most attention (i.e., fixational *dwell time*). Furthermore, processes of resolving the tension created by the cut and of filling in the gap (opened up by first-pass reading) were expected to give rise to a pattern of re-reading (i.e., second- and third-pass reading) eye movements characterized by regressive and progressive saccades across the cut (e.g., in L.1-cut haiku, regressions from line 2 or 3 to line 1 and extra progressions from line 1 to line 2 or 3, over and above the first saccade into these lines). This pattern is likely to differ between L.1-cut and L.2-cut haiku, given the differential positioning of the fragment in the first versus the last line: more cross-line re-tracking may be necessary when reading the former poems. Furthermore, the patterns of first-pass and, especially, re-reading (second- and third-pass) eye movements were expected to be influenced by the type of haiku, that is, the functional-conceptualdistance between the juxtaposed parts.

These predictions may appear to be trivial when viewed from a text-based perspective. From this perspective, for instance, the two phrase lines present a longer piece of (relatively) coherent text that would, as such, be read more fluently than the shorter and more remotely related fragment line. This might result in the assignment of a high degree of attention to the fragment line in firstpass reading, when the phrase and fragment are encountered first. Beyond this, however, from a reader-centered, constructivist perspective, readers would have to recognize the two phrase lines as belonging together and being brought into juxtaposition to the fragment line; that is, readers have to establish coherence by understanding (drawing on background knowledge) the difference in connectedness between the two phrase lines and the fragment line. Such processes of achieving closure would predominantly be reflected in eye movements across the cut during *re*-reading (i.e., second- and third-pass reading), for which a (continued) focus on the fragment line would be less trivial. Similar arguments would apply to effects of the cut position, which may differ depending on whether the fragment has been encountered before the phrase and may thus be informing the reading of the phrase, or whether the fragment is encountered after the phrase, perhaps requiring a reinterpretation of the phrase to settle on one (or more) meaning(s) from the multitude of its meaning potential. These arguments would also apply to modulations of the cut effect by haiku type, that is, by the type of functional-conceptual relation between the phrase and fragment parts.

To get at some of these ‘constructive’ processes, we obtained a memory measure in the second, post-reading phase of the experiment in addition to the eye-movement measures. This is in line with recent methodological standards in reading research, which recommend taking into account the process *and* the product of reading (see, e.g. 16 ). The post-reading memory test was not announced to the participants in advance to prevent them from ‘studying’ the haiku presented during the reading phase with a view to having to perform a memory test later on. Consequently, we can be confident that any memory of the haiku read was established purely as a result of participants’ reading the poems for their own understanding, that is, as a result of the mental processes they engaged in when trying to (re-) create the poems’ meaning (rather than employing rehearsal strategies for doing well in the subsequent memory test; for evidence of such optional strategies, see, e.g., 99 ). In the memory-test phase, participants were presented with ‘old’ haiku, that is, haiku they had read in the initial reading phase of the experiment, randomly interspersed with an equal number of foils, that is, ‘new’ haiku they had not read before. The task was to make a yes/no recognition response and, in case of a positive response, rate the certainty associated with this decision: “recollect” with certainty versus recognize as “familiar” with lesser degrees of certainty. This scale was meant to cover the spectrum from explicit, self-aware memory to more implicit (vaguer) feelings of knowing that one has encountered a particular poem before (e.g., 33, 34, 23 ).

Memory performance, in particular when it is associated with recollective experience, can be regarded as a measure of the depth of processing and closure of the meaning Gestalt achieved. For instance, experiencing an ‘aha’ moment as a result of reading might be experienced as rewarding, leading to better consolidation and accessibility of the memory (including recollection of the experience of reading and understanding the haiku) later on. Note that, according to *levels-of-processing* notions (e.g., 18 ), memory performance would be predicted to be (solely) the result of the type of processing directed to the haiku, with ‘deeper’, semantic-elaborative processing (i.e., processing that links what is read to associated knowledge contained in long-term memory) leading to better performance than more ‘shallow’ processing. Accordingly, more complex haiku (requiring a more disfluent processing mode and involving more inferences based on background/text-external knowledge) should be better remembered than simpler haiku (permitting reading in a fluent mode). Also, even if a participant fails to reach an understanding of a haiku after having expended deep, elaborative processing on it, this haiku may still be well remembered (along with recollective experiences), because the failure to achieve closure leaves the tension in place, improving the accessibility of (contents of) the poem (a kind of Zeigarnik effect; 113 ). On the other hand, if the reward and reinforcement deriving from reward is crucial, memory should be better for haiku for which an understanding was actually achieved. To get at some of these moderators of memory performance, in the final (post-memory-test) phase of the experiment, participants rated the haiku they had read in terms of how difficult it was for them to achieve an understanding, and how well they felt they had understood the haiku.

Finally, the study aimed at relating the memory and rating scale measures to the reading mode evidenced in the eye-movement pattern: can memory performance be predicted from the eye-movement patterns?

Briefly noting here the major outcomes: the results showed that eye-movement patterns in initial reading and re-reading are shaped by the structure (position of the cut) and the type (context–action vs. juxtaposition) of the haiku presented, consistent with the idea that reading eye movements can provide insights into the mental processes of poetry comprehension. Furthermore, and in line with this, recognition memory for previously read haiku (with memory performance being regarded as a function of the mental processes engaged in during the re-/reading of the haiku) bears a relationship to aspects of the eyemovement patterns, as well as to the (self-rated) degree of comprehension achieved. Overall, these findings argue in favor of a closer, more comprehensive study of the reading of haiku within the enterprise of neuro-/cognitive poetics, including the full range of neuro-/cognitive methods.

## Method

### Participants

Eleven participants (7 female; mean age: 23.5 years; age range: 18–29 years) volunteered to take part in the study. They were all native speakers of English and (international) students at LMU Munich. They all had normal or corrected-to-normal color vision. All participants were naïve with respect to the precise purposes of the study (beyond those specified in the instruction; see below), and were neither experienced haiku readers nor regular readers of poetry. Participants gave their informed, written consent prior to commencing the experiment and were paid at a rate of 8.00 € per hour.

### Ethical statement

The study was conducted at the Department of Psychology, LMU Munich. All standard experimental procedures involving the collection of purely behavioral data (in the present study: eye-movement record, memory-test responses, and subjective ratings), without requiring any invasive or potentially dangerous methods (which was the case in the present study) are approved by the Department’s Ethics Committee in accordance with the Code of Ethics of the World Medical Association (Declaration of Helsinki). Data were stored and analyzed anonymously.

### Apparatus

**Figure 2. Trial events in the reading phase.**
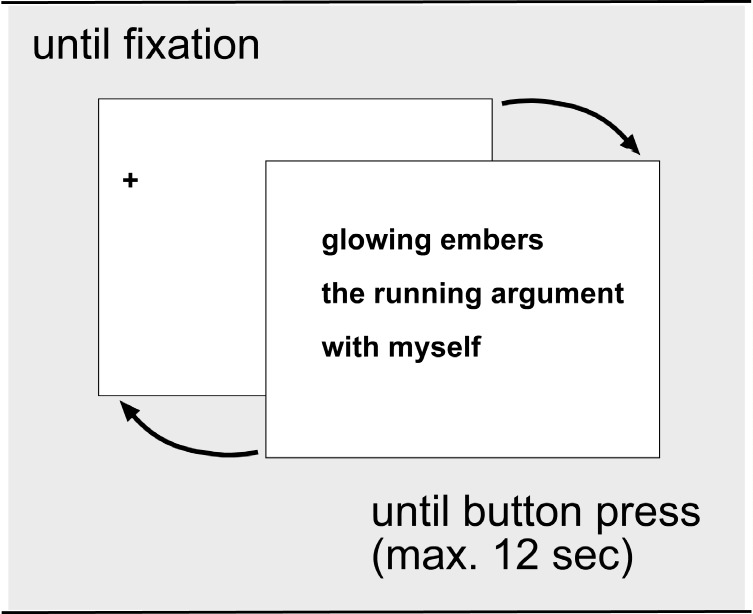
Example display screen, with fixation cross. The poem depicted is by S. Pierides ( 56 ).

The experiment was conducted in a dimly lit and sound-attenuated chamber. The experiment was computer-controlled (standard Intel PC, running XP operating system), with control software purpose-written in C++. Stimuli were presented on a 19-inch CRT monitor (AOC Amsterdam, NL; frame rate: 85 Hz; screen resolution: 1024 x 768 pixels). Participants viewed the monitor from a distance of 63 cm, with head position maintained by a chin-and-head rest device. The haiku to be read during the initial reading phase, all consisting of three lines, were presented *left-aligned* in the center of the monitor (distance: 12.4° from the left margin of the screen). Prior to the onset of the haiku on a given trial, participants were presented with a black (0.5 cd/m²) fixation marker, a cross symbol (0.4° of visual angle), to the left of (the left-side boundary of) the first word in line 1; the distance between the cross and first word was 0.8°. Overall, given the viewing distance of 63 cm, the average haiku covered a screen area of some 4.5° x 8.5° of visual angle (letter type: Arial; letter size: 0.72°; line spacing: 0.73°; font color: black, 0.5 cd/m²; display background: white, 30.0 cd/m²); note that the exact measures varied with the line lengths (in terms of word/syllable/letter numbers per line) among the various haiku, while not differing significantly between the four type of haiku x cut position conditions. See Figure 2 for an example display screen. During reading, participants’ eye movements were recorded, at a sampling rate of 1000 Hz, using a remote SR Research EyeLink 1000 desktop-mount eye-tracker (SR Research Ltd., Mississauga, Ontario, Canada). Sampling on a given trial was started by the experimenter (by pressing the space key on a standard German keyboard on the control computer) as soon as stable fixation on the fixation marker (defined as the eye resting approx. 1 sec on the cross symbol) was established, and ended either once the participant indicated (by pressing the cursor-down key on the display computer keyboard) that she/he had completed reading or else after the maximum haiku reading (=presentation) time of 12 sec. The recording was calibrated prior to the reading, and calibration accuracy was checked by the experimenter, who manually started haiku presentation only when the participant was seen to gaze at the fixation cross (no re-calibration was carried out during the relatively short, 15-min experiment). During the subsequent memory-test phase, participants were again presented with the full set of haiku on the screen (those read as well as unread foils), and had to give (i) a yes/no recognition response and (ii), in case of a positive response, a five-point scale rating of the certainty associated with this response to each haiku (a standard procedure in recognition memory research; see, e.g. 33, 34, 23 ). The yes/no response was made using the <y> and, respectively, <n> keys on the keyboard placed on the table in front of the participant, and the ratings using the numerical keys <1> through <5> – the specific question being: “How certain are you that you have seen this haiku earlier on? (1 = ‘I definitely recollect having seen the haiku’, and 2–4 = ‘I feel I have seen the haiku’)”, with various (degrees of) strengths associated with this ‘feeling of familiarity’. The questions were presented in green and red color (recognition and certainty question, respectively) at the top of the screen (distance from top screen margin: 4.32°), covering a screen area of about 2.7° x 16.0° of visual angle (letter type: Arial; letter size: 0.57°; line spacing: 0.50°; green font: 8.0 cd/m²; red font: 7.7 cd/m²). The memory response and associated certainty ratings were stored on the display computer (along with an identifier of the haiku tested). In the final phase of the experiment, participants were re-presented with the haiku they had actually read, and they had to rate each haiku in terms of how difficult a given haiku was to understand (scale: 1–5; 1=very easy, 5=very hard; font color: green) and whether they had achieved an understanding of the haiku’s meaning (scale: 1–5; 1=full understanding, 5=no understanding; font color: red). Again, these subjective rating data were stored for later analysis.

### Materials

The ELH poems to be read by the participants, 48 haiku in total, and the foils additionally presented during the memory test (another 48 haiku), were selected from highly reputed (English-language) haiku journals and registries (such as *Frogpond*, *Modern Haiku*, *The Heron’s Nest*, *A Hundred Gourds*, *The Haiku Foundation*, among others) by the co-authors. All selected poems were three-line haiku, and each 50% of the poems were, in terms of the classification proposed by Kacian ( 49 ), *‘context-and-action haiku’* (in brief, *context–action haiku*, in which the fragment provides the setting and the phrase an activity within this context) and, respectively, ‘haiku of *juxtaposition*’ (*juxtaposition haiku*, in which the fragment and phrase images are related in some other, more remote way). See Figure 1 for examples (examples reprinted with written permission of the authors). Furthermore, all haiku had a clearly discernible cut (agreed by the co-authors), either after line 1 (L.1-cut haiku) or after line 2 (L.2-cut haiku); see illustrations in Figure 1. This resulted in four sets of haiku or experimental conditions: context–action L.1-cut and L.2-cut haiku and juxtaposition L.1-cut and L.2-cut haiku. Post-selection analyses (line x cut position x haiku type) ensured that these four sets were overall not significantly different in terms of number of letters, syllables and words per line, number of morphemes and phrases, ratio of content to function words (and thus to words which tend to be skipped by readers; e.g., 24, 12, 84 ), (variation in) position and form of realization (finite, infinite, ellipted) of the verb (as the central valency carrier and thus determinant of sentence structure; e.g., 41 ), (frequency and context of) occurrence of phoric elements like pronouns or definite determiners (the identification of whose antecedents has been reported to result in longer fixation durations and/or regressive saccadic movements; e.g., 24, 11, 76 ), (frequency of) occurrence of potentially attention-attracting stylistic features like alliterations, (sentence- and phraseinternal) enjambements (e.g., 61 ), unusual syntactic patterns (i.e., word order other than SVO), and (frequency of occurrence of) low-frequency words and (two-word) collocations (occurrence: <1/million words in the British National Corpus, 5 ; frequency data were calculated using Sketchengine; see 56, 55; Information on effects of word frequency on eye movements during reading can e.g., be found in 89, 94, 85. For all (3 [line] x 2 [haiku] x 2 [cut position] repeated-measures ANOVA) tests: ps > .37, BFs > .94. The only significant effect revealed was the content-tofunction word ratio for line 2 (chi²=12.28, p < 0.01, BF=87), which was somewhat increased for L.1-cut context–action haiku; as this effect was not reflected in the eye-movement results, we will not consider it any further). Given the absence of relevant differences with respect to these (linguistic) variables, it can be considered unlikely that any of the effects reported in the results are attributable to them.

Note that, for the present study, we opted not to present the participants with any ‘control’ texts to the haiku they read for two reasons: (i) approaching a text in a ‘poetic’ attitude of reading (having been instructed that the texts are poems) differs fundamentally from the reading of ordinary text (see, e.g. 10, 40, 111, 112 ); and (ii) it is hard to agree on what would actually constitute an appropriate control text. Concerning the latter, for instance, in what sense would, say, a syntactically regularized, ‘uncut’ sentence (without line breaks) re-describing a haiku using (much) the same words provide a suitable control (e.g., “As they cross the border at night, the elephant calf holds his mother's tail”)? Note that such re-descriptions would not always be possible (especially for juxtaposition haiku) because the haiku's juxtaposed parts may ‘refuse’ to be brought together in a regular English sentence – quite apart from the fact that in most cases such sentences would require the use of relatively more free grammatical morphemes (e.g., prepositions, determiners, conjunctions, etc.), which would result in the loss of the brevity and punchiness characteristic of haiku. Merely removing the line breaks while retaining the irregular and/or fragmentary syntactic structure does not constitute an option either. As reported by Yaron ( 112 , p. 132) such poembased texts are usually rejected by readers as unacceptable and/or incomprehensible, because they do no trigger the specific mode of ‘poetic’ reading which renders readers willing to accept and deal with seemingly obscure, formally and/or semantically highly irregular forms of language use.

### Design and Procedure

The experiment varied two (main) variables in an orthogonal manner: type of haiku (context– action, juxtaposition) and cut position (L.1-cut, L.2-cut). The experiment consisted of three distinct phases, after the initial instruction: (i) reading, (ii) memory test, and (ii) subjective ratings.

In the reading phase, the very same haiku (belonging to the four sets) were presented to all participants in a trial order determined randomly on an individualparticipant basis (i.e., all forms of haiku were presented in completely randomized order, rather than blocked according to haiku type or cut position). Each haiku was presented for a maximum time of 12 sec, or shorter if the participant terminated reading (by pressing the cursordown key) before this deadline. Following a blank interval of 1 sec, the next trial started automatically with the fixation marker. Participants were instructed to “read each haiku attentively for your own understanding, trying to recreate the images presented in your mind. Your eye movements will be recorded while you read the haiku” (see supporting material S2 for the full instruction).

At the end of the reading phase (which lasted about 15 minutes in total), participants were given a rest period of some 3 minutes (in which they stayed in the experimental room). Subsequent to this, participants were informed that, in the next phase, they would be presented with haiku they had already read as well as new haiku they had not seen before; the task was to respond “yes” to each haiku they recognized as ‘old’ (and, respectively, to respond “no” to ‘new’ haiku); a yes-response was immediately followed by the question: “How certain are you that you have seen this haiku earlier on? 1 = “I definitely recollect having seen the haiku” and 2–4 = “I feel I have seen the haiku”, with various (degrees of) strengths associated with this ‘feeling of familiarity’. In this memorytest phase, read haiku and foils were presented in random order (with the order of read haiku itself randomized; that is, it differed randomly from the order in which these haiku were initially encountered).

The final, subjective-rating phase followed immediately afterwards. In this phase, participants were represented – and explicitly told so – only with the haiku they had actually read in the first phase of the experiment (with a new, randomized order in phase 3) and were asked to indicate the following: “How difficult was this haiku to understand?” (scale: 1=very easy – 5=very difficult) and “Did you achieve an understanding of this haiku?” (scale: 1=fully understood – 5=completely failed to understand). At the end of phase three, participants were debriefed: apart from gathering information about whether they were or were not familiar with the genre of haiku poetry, they were given more information about this form of poetry (including an information sheet with a brief explanation and web-links for further reading) and more details about the purpose of the study.

Altogether, these three phases (plus debriefing) took about 50 minutes to complete.

### Analysis

Data analyses were performed using R ( 82 ). Both Frequentist and Bayes statistics were computed. Bayes Factors were calculated using the R package “BayesFactor”( 75 ). Unless stated otherwise, analyses of variance (ANOVAs) were repeated-measures (rm) ANOVAs with the factors haiku type (context–action, juxtaposition), cut position (L.1-cut, L.2-cut), and line (1, 2, 3.)

Eye-movement analysis. The eye-movement record was stored and later on analyzed off-line with purposewritten C++ software. For this, we defined three different rectangular ‘regions-of-analysis’ (ROA) areas (size: 10.63° x 1.66°) positioned on top of the three poem lines, with identical, display-centered coordinates for each observer: ROA 1 was positioned at x-y coordinates 12.35°-10.21°, ROA 2 at 12.35°-11.90°, and ROA 3 at 12.35°-13.59°, with a vertical separation of 0.03° between adjacent ROAs. Only fixations that fell in any of the three ROAs were considered for further analysis. This led to the loss of some 4.5% of all fixations. Saccades were separated from fixations based on standard velocity and acceleration criteria (i.e., the SR Research default settings: velocity exceeding 35°/sec and acceleration exceeding 9500°/sec²). The x-y coordinates of a given fixation were determined by averaging the x-y coordinates across all 4-ms (i.e., 250-Hz sampling frequency) time bins during the duration of a given fixation.

The first saccade was defined as the first eye movement landing 0.8° to the right of the fixation cross. Only 13.7% of the trials were automatically terminated when reading time exceeded 12 sec (timed-out trials); all other trials were terminated manually (with a button press) by the participants after an average reading time of some 6.5 seconds (6311 ms). Both timed-out and manually terminated trials were included in the analyses.

**Figure 3: Scan paths produced by one participant for two of the haiku read.**
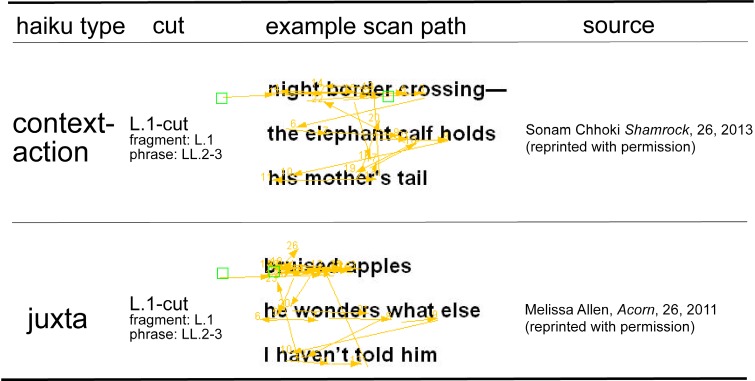


Given the complexity of the reading scan paths (see Figure 3 below for examples), our approach was to look at general eye-movement patterns that describe whole (haiku type x cut position) categories of poems in summary terms. We did this in two stages:

In stage 1, the eye-movement records were analyzed ‘globally’, in terms of the dwell times (aggregated across fixations) and the number of fixations per line, related – or ‘normalized’ – to the number of words per line [Note 1]. The latter was necessary to equate for unequal numbers of words per line (as a rule, there were more words in line 2 than in lines 1 and 3).

Stage 2 was meant to reveal a more detailed picture of the reading eye-movement dynamics (‘saccadic activity’), by examining the sampling of the haiku in terms of the first-, second-, and third-pass reading of each line – where a reading pass starts with the eye (re-)entering a given line and ends with the eye leaving this line. (We desisted from analyzing reading passes beyond pass number 3, as there were insufficient – 4th- etc. pass – data for statistical examination.)

In particular, in this stage, we were interested in the interplay between forward- and backward-directed eyemovement activity over the course of reading. To get at these dynamics, we analyzed, separately for each reading pass, the progressive and regressive eye movements – that is, saccade probabilities and post-saccadic dwell times, both normalized per word – as a function of the haiku type and cut position for each line of the poems. For these analyses, saccades and post-saccadic dwell times were classed as *regressive* when the eye was directed leftwards, from a given position (more to the right), within a line (*intra-line regression*) or from a lower to a higher line (*cross-line regression*, e.g., from line 3 to line 2 or line 1) [Note 2]. Conversely, saccades and post-saccadic dwell times were classed as *progressive* when the eye was projected rightwards, from a given position (more to the left), within a line (*intra-line progression*) or from a higher to a lower line (*cross-line progression*, e.g., from line 1 to line 2 or 3). It should be noted that in this line-based way of analyzing (re-)reading eye movements, information is lost as to when, or in which sequence, exactly a given line was (re-)entered. However, we can at least say something about the rank order in which particular lines were (re-)visited in the various reading passes.

Analysis of recognition memory. For recognition performance, only haiku that received a correct recollection or familiarity response were considered for analysis. This involved the removal of 14% of the –‘missed’ – haiku.

## Results

The results will be presented in two sections: eyemovement analyses and subjective-rating analyses (recollection, haiku difficulty, understanding achieved), respectively.

### Eye-movement results

Overall, looking at the scan paths of our readers (see Figure 3 for two examples), it is clear that reading haiku involves a complex, non-linear pattern of eye movements: readers go forward and backward within lines, and they jump between lines not only in the standard, forward path, but they also go back, for instance, from the end to beginning of the poem (see also 111, 112 for similar findings for other types of poems). Thus, frequently, a poem is sampled not only once, but two or three times. Importantly, re-reading usually does not involve a ‘straight’ path (e.g., the eye may return to line 1 via line 2 from line 3 and then jump directly to line 3 from line 1), thus reflecting complex and non-linear processes of visual information gathering and meaning construction. Given the complexity of the scan paths (which differ between individual poems and readers), our approach was to look at general eye-movement patterns that describe whole (haiku type x cut position) categories of poems in summary terms. What we outline below are analyses and results based on these summary measures.

#### Analysis of total fixation probabilities and dwell times

In the first instance, the eye-movement records were analyzed ‘globally’, in terms of the dwell times (aggregated across all fixations) and the number of fixations per line. The data are presented in the top half of Table 1, as a function of haiku type (context–action, juxtaposition) and cut position (L.1, L.2), for the three lines of the poems. As can be seen, overall, the (total) dwell time and number of fixations are increased for line 2 relative to lines 1 and 3. However, this pattern is ‘confounded’ by the differential line lengths: the middle line is typically longer, that is, it contains more words (letters, syllables, morphemes, etc.), than the first and the third line (in terms of words: 3.49 vs. 2.31 and 2.72, respectively). To correct for this difference and make the values comparable across lines, the lower half of Table 1 presents the same data related to the number of words in the various lines. Recall that the haiku in the various haiku type x cut positions conditions did not differ in word length (per line), whether measured in terms of the number of letters or syllables (see Stimulus Materials above). Accordingly, all statistical effects reported below for ‘words per line’ would also be significant if related to, say, ‘number of letters per line’.

**Table 1:** Dwell times in ms [number of fixations in parentheses].

	**L.1 c-a**	**L.2 c-a**	**L.1 juxta**	**L.2 juxta**
**Line 1**	1470 [4.79]	985 [3.09]	1697 [5.09]	1677 [5.58]
**Line 2**	2098 [7.96]	2001 [7.09]	2038 [7.58]	2101 [7.95]
**Line 3**	1474 [4.92]	1763 [5.55]	1845 [5.80]	2027 [6.53]
Line 1*	668 [2.18]	657 [2.06]	757 [2.27]	508 [1.69]
Line 2*	567 [2.15]	666 [2.36]	576 [2.14]	567 [2.15]
Line 3*	566 [1.89]	704 [2.21]	489 [1.54]	1024 [3.27]

Note: * values per word (correcting for differential line lengths in terms of no. of words). Fisher Least Square Difference = 210 ms [likelihood of fixations, Fisher Least Square Difference = .52].

As can be seen, the mean dwell time per word is longer in the fragment line (line 1 in L.1-cut haiku; line 3 in L.2-cut haiku) compared to the other (phrase) lines: 768 ms vs. 575 ms. This difference is larger for haiku with a cut after line 2 (L.2-cut haiku: line 3 vs. lines 1–2: 859 ms vs. 599 ms) relative to haiku with a cut after line 1 (L.1-cut haiku: line 1 vs. lines 2–3: 712 ms vs. 549 ms) and essentially similar for the two haiku types, though the extended dwell time per word in the (fragment) line before and, respectively, after the cut (relative to the other lines) is particularly marked (and, given the Fisher Least Square Difference provided in Table 1, statistically significant only) for juxtaposition haiku (juxtaposition: 886 ms for the fragment line (line 1 or 3) and 535 ms for the phrase lines (i.e., lines 2 and 3 or lines 1 and 2); context: 686 and 614 ms). These observations are substantiated by a 2 (haiku type: context–action, juxtaposition) x 2 (cut position: L.1, L.2) x 3 (line: 1, 2, 3) rm ANOVA which revealed the haiku type x cut position x line interaction to be significant: F(2,10)=5.34, p<.01, BF=5.18. Thus, the analysis of total fixational dwell times discloses extended processing of the (fragment) line before (L.1-cut haiku) and, respectively, after the cut (L.2-cut haiku), with this pattern being more marked (i.e., statistically significant only) in juxtaposition haiku. [Note 3]

#### Analysis of first-, second-, and third-pass reading

To gain a more detailed picture of the reading eyemovement dynamics (saccadic activity), we went on to examine the sampling of the haiku in terms of the first-, second-, and third-pass reading of each line. Each line was entered (and read) as least once in all conditions (100% overall), with decreasing probabilities of entering (i.e., re-reading) a line for a second time (65%) or three (41%) or more times. In terms of how frequently a given line was re-entered in the second and third pass (see Supplementary Table 1), there was little difference among the various lines (2nd pass, line 1 vs. line 2 vs. line 3: 62% vs. 73% vs. 60%; 3rd pass: 37% vs. 51% vs. 35%); also, there were no differences in the rates of re-reading among the four haiku type x cut position conditions (2nd pass, L.1-cut context–action vs. L.2-cut context–action vs. L.1cut juxtaposition vs. L.2-cut juxtaposition: 66% vs. 65% vs. 66% vs. 62%; 3rd pass: 39% vs. 44% vs. 40% vs. 40%). Statistically, line 2 was somewhat more likely to be revisited, relative to lines 1 and 3, in both the second pass (12% increase, F(2,20)=3.56, p<.05, FLSD=.10, BF=.94) and the third pass (15% increase, F(2,20)=6.89, p<.01, FLSD=.10, BF=2.55). There may be two reasons for this: (i) line 2 is ‘on the way’ back from line 3 to line 1 (i.e., the eye may stop briefly in line 2 on its way to line 1), and forward from line 3 to line 1, and (ii) it contains, on average, more words than lines 1 and 3 (in fact, related to the number of words, line 2 is not more likely to be revisited).

Of particular interest for understanding processes of meaning construction is the interplay between forwardand backward-directed eye-movement activity over the course of reading – with the re-reading of sections of text, or words, already read being, arguably, particularly indicative of meaning clarification and resolution processes (e.g. 111 ). The data summarized in Tables 2, 3, and 4 present, separately for each reading pass, the analyses of progressive and regressive eye movements (saccade probabilities [values in square parentheses] and postsaccadic dwell times, both normalized per word) as a function of the haiku type and cut position for each line of the poems.

While first-pass reading was characterized by a linear progression from line 1 through line 2 to line 3 (i.e., 1-2-3 rank order of lines), second-pass reading might have started with either line 1 or line 2, and then all sorts of sequences (including an interspersed third pass at one or two particular lines, e.g., 1-2-**1**-3 or 1-2-**1**-**2**-3 [2nd pass in italics, 3rd pass in bold]) were possible. Given that some of our participants were ‘rare’ re-readers and the rereading rates differed between poems, our current sample is too limited to permit such a fine-grained, sequencebased analysis. However, we can at least say something about the rank order in which particular lines were revisited in second- and third-pass reading: second-pass rereading was equally likely to start with line 1 or line 2 (average ranks of 1.52 and 1.48, respectively), and line 3 was re-entered following lines 1 or 2 or both lines 1 and 2 (average rank of 2.46) – a pattern that was seen in all haiku type x cut position conditions; Friedman (one-way ANOVA) tests on the rank-order data confirmed this pattern to be significant for all four conditions (see Supplement Table S2 for the full data set). This priority for lines 1 and 2 relative to line 3 is also evident in the third pass (average ranks of 1.49, 1.48, and 2.02 for lines 1, 2, and 3 respectively), though it is less marked, owing to the greater variability in when the third pass occurred for a particular line (see above). Again, Friedman tests revealed this pattern to be significant for all (haiku typex cut position) conditions, except for L.2-cut context-action haiku for which the ranks were statistically indistinguishable among the three lines (1.81, 1.64, and 2.02 for lines 1, 2, and 3, respectively; Friedman χ² = 2.95, p=.23). [In addition, for the third pass, line 2 had (some modest) priority over line 1 for all (haiku type x cut positions) conditions – except for L.2-cut juxtaposition haiku for which re-reading was highly likely to start with line 1 (Friedman χ² = 13.91, p<.001; ranks: 1.18 vs. 1.61 and 1.95 for lines 1, 2, and 3, respectively; the priority of line 1 over line 2 (and line 3) was substantiated by a – Wilcoxon signed-rank – test: p<.05). Note that the (significant) Friedman χ²-values (range: 8.95 to 16.54) were significant even when using relatively conservative alpha-levels (.01 or .001), to prevent inflation of type-I errors.

Despite the limited information we can extract regarding the exact order in which lines were read or re-read, looking at the eye-movement data for each line averaged across all possible cross-line transitions is nevertheless informative as it provides summary reading (‘fluency’) parameters for when a given line was entered for the first, the second, and the third time.

Looking at Tables 2, 3, and 4, what becomes immediately apparent is that the overall dwell time per word decreases with the number of reading passes (aggregated across pro- and re-fixations: 292 [pro 200, re 92] ms, 149 [76, 73] ms, and 120 [75, 45] ms for the first, second, and third pass, respectively). A 2 x 3 repeated-measures ANOVA comparing the dwell times between pro- and refixations across the three reading passes revealed the interaction to be significant (besides a significant main effect of pass: F(2,20)=22.57, p<.001, BF=1.7+e5): F(2,20)=16.17, p<.01, BF=5.3+e9, FLSD: 28 ms. Profixations showed a marked decrease in dwell times from the first to the second pass and then remained stable (200 ms vs. 76 and 75 ms). Re-fixations, by contrast, exhibited a dwell-time decrease only from the second to the third pass (73 vs. 46 ms), while being statistically comparable between the first and the second pass (92 vs. 73 ms).

This illustrates that reading a poem (line) a second or third time (or even more times) is increasingly ‘selective’, probably serving to check interpretations generated, or fill-in gaps left open, on the preceding pass(es).

##### First-pass reading

As can be seen from Table 2A, in the first pass, *progressive* saccades are more frequent within the fragment line (.79) than within the phrase lines (.61, averaged across the two phrase lines), relatively independently of the haiku type and cut position. L.2-cut context–action haiku deviate from this general effect in one respect: profixations are more frequent in the first phrase line (line 1) than in the fragment line (line 3). Generally mirroring the focus on the fragment line, the aggregated dwell times (per word) following progressive saccades are overall longer in the fragment line (246 ms) than in the phrase lines (177 ms, averaged across the two phrase lines). This pattern is most clearly seen with juxtaposition haiku (251 vs. 157 ms). For context–action haiku, by contrast, the dwell times are as long in the first phrase line (i.e., line 2 in L.1-cut haiku and line 1 in L.2-cut haiku) as in the fragment line. These differential patterns are reflected in significant haiku type x cut position x line interactions (pro-fixation probability: F(2,20)=9.00, p<.01, BF=28.55; *dwell times* : F(2,20)=10.38, p<.01, BF=17.55).

As can be seen from Table 2B, *regressive* saccades are most likely within line 3 (.54), compared to lines 1 and 2 (.14 and .24, respectively) – for all (haiku type x cut position) conditions. Likewise, the dwell times (per word) following regressive saccades are longest in line 3, compared to lines 1 and 2 (167 ms vs. 38 ms and 70 ms, respectively). This dwell-time effect is more pronounced for L.2-cut haiku, where the third line is the fragment line, compared to L.1-cut haiku (202 ms vs. 132 ms), with a particularly marked difference between L.2-cut and L.1-cut juxtaposition haiku (231 ms vs. 110 ms). This pattern is statistically substantiated by significant haiku type x cut position x line interactions (re-fixation probability: F(2,20)=14.15, p<.01, BF=18.42; *dwell times*: F(2,20)=5.61, p<.01, BF=7.55).

In summary, in the first pass, scanning is predominantly forward-directed (in all lines) and focused on the fragment line in both context–action and juxtaposition haiku, as well as on the first phrase line in context–action haiku. Within-line regressions, which are relatively infrequent in lines 1 and 2 (regression vs. progression probability: .18 vs. .70; dwell time: 54 vs. 199 ms), are concentrated on the third line in all conditions, with re-fixation probability and dwell time approaching pro-fixation probability and dwell time (.54 vs. .61; 167 ms vs. 203 ms). In other words, while lines 1 and 2 are processed relatively fluently (in a predominantly forward-directed scan), reading is more disfluent (involving increased backward-directed scanning) in line 3. In L.2-cut haiku generally, and especially in L.2-cut juxtaposition haiku, the final-line re-fixations add substantially to the profixations, yielding what (in the data aggregated across pro- and re-fixations) manifests as a very marked ‘dwell’ on the fragment line.

**Tables 2, 3, and 4.** Fixational dwell time (per word in ms) following progressive and regressive saccades, for each of the three lines, in first-pass (2A, 2B), second-pass (3A, 3B), and third-pass reading (4A, 4B) of the various lines, separately for each haiku type x cut position condition. The numbers in square parentheses (i.e., []) give the likelihood with which a word in a given line is fixated following a progressive or a regressive saccade in first-, second-, and thirdpass reading, respectively. (Note that, because we did not analyze reading passes beyond the 3rd reading, the dataset in which Tables 2, 3, and 4 are based is smaller than the full dataset which forms the basis of Table 1).

**Table 2A:** First-pass dwell times (per word) following progressive saccades, Fisher Least Square Difference = 77 ms [likelihood of pro-fixations, Fisher Least Square Difference = 0.11].

**1st pass**	**L.1 c-a**	**L.2 c-a**	**L.1 juxta**	**L.2 juxta**
**Line 1**	244 [0.81]	263 [0.88]	232 [0.82]	155 [0.62]
**Line 2**	239 [0.63]	134 [0.61]	164 [0.58]	158 [0.66]
**Line 3**	153 [0.50]	239 [0.71]	151 [0.43]	270 [0.80]

**Table 2B:** First-pass dwell times (per word) following regressive saccades, Fisher Least Square Difference = 30 ms [likelihood of re-fixations, Fisher Least Square Difference = 0.08].

**1st pass**	**L.1 c-a**	**L.2 c-a**	**L.1 juxta**	**L.2 juxta**
**Line 1**	42 [0.17]	15 [0.06]	57 [0.17]	39 [0.16]
**Line 2**	84 [0.32]	48 [0.12]	75 [0.25]	74 [0.28]
**Line 3**	153 [0.55]	172 [0.47]	110 [0.35]	231 [0.78]

**Table 3A:** Second-pass dwell times (per word) following progressive saccades, Fisher Least Square Difference = 44 ms [likelihood of pro-fixations, Fisher Least Square Difference = 0.14].

**2nd pass**	**L.1 c-a**	**L.2 c-a**	**L.1 juxta**	**L.2 juxta**
**Line 1**	69 [0.22]	48 [0.15]	69 [0.21]	39 [0.12]
**Line 2**	65 [0.27]	64 [0.21]	64 [0.22]	101 [0.27]
**Line 3**	59 [0.21]	146 [0.38]	70 [0.20]	120 [0.46]

**Table 3B:** Second-pass dwell times (per word) following regressive saccades, Fisher Least Square Difference = 44 ms [likelihood of re-fixations, Fisher Least Square Difference = 0.10].

**2nd pass**	**L.1 c-a**	**L.2 c-a**	**L.1 juxta**	**L.2 juxta**
**Line 1**	108 [0.32]	122 [0.36]	105 [0.31]	56 [0.16]
**Line 2**	45 [0.19]	37 [0.15]	58 [0.19]	42 [0.17]
**Line 3**	80 [0.24]	47 [0.20]	51 [0.15]	120 [0.33]

**Table 4A:** Third-pass dwell times (per word) following progressive saccades, Fisher Least Square Difference = 52 ms [likelihood of pro-fixations, Fisher Least Square Difference = 0.07].

**3rd pass**	**L.1 c-a**	**L.2 c-a**	**L.1 juxta**	**L.2 juxta**
**Line 1**	29 [0.09]	148 [0.21]	36 [0.12]	66 [0.11]
**Line 2**	53 [0.21]	67 [0.12]	55 [0.18]	103 [0.21]
**Line 3**	33 [0.11]	135 [0.24]	33 [0.08]	141 [0.24]

**Table 4B:** Third-pass dwell times (per word) following regressive saccades, Fisher Least Square Difference = 31 ms [likelihood of re-fixations, Fisher Least Square Difference = 0.09].

**3rd pass**	**L.1 c-a**	**L.2 c-a**	**L.1 juxta**	**L.2 juxta**
**Line 1**	66 [0.17]	77 [0.24]	58 [0.15]	32 [0.08]
**Line 2**	32 [0.12]	44 [0.12]	39 [0.12]	41 [0.13]
**Line 3**	33 [0.10]	36 [0.13]	24 [0.07]	63 [0.17]

##### Second- and third-pass reading

While *forward-directed* scanning in the first pass exhibited a focus on the fragment line in all conditions, only L.2-cut haiku exhibit such a pattern – of an increased probability of pro-fixations and prolonged dwell times on words within the fragment line – in the second and third pass (see Tables 3A and 4A; fragment vs. phrase lines, 2nd [2nd+3rd] pass: pro-fixation probability: .42 [.33] vs. .19 [.18]; dwell time, 133 [136] ms vs. 63 [80] ms). In L.1-cut haiku, by contrast, second- and third-pass scanning activity is relatively balanced across the fragment and phrase lines (2nd [2nd+3rd] pass: probability: .22 [.16] vs. .23 [.19]; dwell time, 69 [51] ms vs. 65 [54] ms). For second-pass reading, this differential (fragment vs. phrase line) pattern is reflected in significant cut position x line interactions (probability: F(2,20)=13.14, p<.01, BF=18.08; *dwell times*: F(2,20)=10.43, p<.01, BF=8.76). Note that in L.2-cut juxtaposition haiku, besides the primary focus on the fragment line, there is a secondary focus of progressive re-sampling on the second phrase line (line 2), in both the second and the third pass (1st phrase vs. 2nd phrase vs. fragment line, 2nd+3rdpass: probability: .12 vs. .24 vs. .35; dwell time: 53 ms vs. 102 ms vs. 131 ms) – a pattern not seen in the first pass. Furthermore, in the third pass, L.2-cut context–action haiku exhibit a pattern first seen in first-pass (but not seen in second-pass) reading: the first phrase line (line 1) again receives as much re-scanning activity as the fragment line (line 3) (1st phrase vs. 2nd phrase vs. fragment line: probability: .21 vs. .12 vs. .24; dwell time: 148 ms vs. 67 ms vs. 135 ms).

Also different to *backward-directed scanning* in the first pass (which was concentrated on line 3 in all conditions), in second- and third-pass reading (see Tables 3B and 4B), most regressive saccades occur within and/or are directed to the fragment line (i.e., line 1 in L.1-cut haiku and line 3 in L.2-cut haiku): 2nd- [2nd+3rd-] pass refixation probabilities of .29 [.23] (fragment line) versus .20 [.16] (phrase lines combined). The 2nd- [2nd+3rd-] pass dwell times show a similar pattern: 95 [81] ms (fragment line) versus 52 [46] ms (phrase lines combined). While this pattern is clear for (both L.1- and L.2-cut) juxtaposition haiku, with context–action haiku it is seen only for L.1-cut, but not L.2-cut haiku: for the latter, regressive activity is focused on the first phrase line (line 1), rather than the fragment line (line 3) (2nd- [2nd+3rd-] pass, 1st phrase vs. fragment line: probability, .36 [.30] vs. .20 [.17]; dwell time, 122 [100] vs. 47 [42] ms). This pattern is substantiated by significant haiku type x cut position x line interactions for both the second pass (re-fixation probabilities: F(2,20)=8.27, p<.01, BF=9.98; re-fixation dwell times: F(2,20)=8.28, p<.01, BF=9.88) and the third pass (re-fixation probabilities: F(2,20)=3.59, p<.05, BF=3.96; re-fixation dwell times: F(2,20)=3.23, p=.06, BF=3.65).

Looking at the combined, forward-directed and backward-directed scanning activity, some more global patterns – distinguishing L.1-cut from L.2-cut haiku generally, and L.2-cut context–action from L.2-cut juxtaposition haiku specifically – become discernible.

For *L.1-cut haiku* (whether of the context–action or the juxtaposition type), the re-reading pattern is relatively straightforward to characterize: in the second pass, there is extensive re-sampling of the fragment line (line 1), with more regressive than progressive activity within this line (re- vs. pro-fixation probability: .32 vs. .22; dwell time: 107 ms vs. 69 ms) – indicative of a disfluent mode of reading. By comparison, there is only little re-sampling of the phrase lines (with a relative balance of regressive and progressive movements: probability: .19 vs. .23; dwell time: 59 vs. 65 ms) – indicative of a more fluent scanning of these lines. This pattern essentially repeats in the third pass, though this time with a less marked focus on the fragment line (fragment line re- vs. pro-fixation probability, .16 vs. .11; dwell time, 62 ms vs. 33 ms).

Differential patterns of re-reading emerge between L.2-cut context–action and L.2-cut juxtaposition haiku.In *L.2-cut context–action haiku*, second-pass re-sampling is focused on the first phrase line, with a dominance of reover pro-fixations within this line (probability: .36 vs. .15; dwell time: 122 ms vs. 48 ms) – indicative of a disfluent reading mode. There is then a renewed focus on the fragment line (i.e., line 3; rather than one to the second phrase line, i.e., line 2), where pro-fixations dominate re-fixations (probability: .38 vs. .20; dwell time: 148 ms vs. 47 ms) – indicative of a more forward-directed scanning. Third-pass re-sampling exhibits a similar pattern, though scanning is now predominantly forwarddirected (rather than backward-directed) in both the first phrase line (pro- vs. re-fixations: probability: .21 vs. .24; dwell times: 148 ms vs. 77) and the fragment line (.24 vs. .13; 135 ms vs. 36 ms).

In *L.2-cut juxtaposition haiku*, by contrast, secondpass reading is characterized by a focus on the second (rather than the first) phrase line, with a dominance of pro- over re-fixations within this line (probability: .27 vs. .17; dwell time: 100 ms vs. 42 ms), indicative of relatively fluent, forward-directed scanning. There is then a renewed focus on the fragment line (line 3), with a dominance of progressive over regressive movements but balanced dwell times (probability: .46 vs. .33; dwell time: 120 ms vs. 120 ms). This pattern essentially repeats in the third pass, now with a dominance of forward- over backward-directed activity in both the second phrase line (probability: .21 vs. .13; dwell time: 103 ms vs. 41 ms) and the fragment line (.24 vs. .17; 141 ms vs. 63 ms).

Overall, it would appear that third-pass reading is more fluent than second-pass reading, consistent with the idea the second-pass reading may generate resolution hypotheses (especially in lines where re-reading involves a large proportion of regressive scanning) that are (just) re-checked in the third pass (see Discussion for an elaboration of this proposal).

It should be noted that the result pattern, and dynamics, revealed in the above analyses does not change when we look at the second- and first-pass sampling only for those haiku (or lines) that were read at least three times (i.e., some 45% of the haiku, or lines; see Supplement Tables S3 and S4 for the full dataset). This indicates that the first- and second-pass reading dynamics stays essentially the same irrespective of whether a haiku (or line) is read a second or third time.

#### Memory and Subjective Rating Results

Overall, memory performance was remarkably high: 86% of all haiku were correctly recognized in the memory test phase as having been read before (‘hits’). In other words, there were only 14% of recognition failures (‘misses’) – likely owing to the fact that the recognition task was too easy for our (relatively young, adult) participants. Furthermore, the great majority of correctly recognized haiku was associated with participants reporting ‘recollective experience’ of having encountered the respective haiku before (70%), and only a small portion (16%) with a ‘feeling of familiarity’ in the absence of recollective experience.

Of note, the ratio between the numbers of poems that yielded a recollection versus a familiarity response was not systematically influenced by haiku type: 6.70/1.0 for context–action and 4.84/1.0 for juxtaposition haiku (twotailed t test: t(10)=.86, p=.41, BF=.40). Accordingly, there was no evidence that the differential patterns of reading eye movements that characterize the two haiku types are associated with different types of recollective experience. Interestingly, an analogous comparison for the factor of cut position revealed the proportion of recollection responses to be reliably higher for L.1-cut relative to L.2-cut haiku (7.31/1.0 vs. 2.77/1.0, two-tailed t test: t(10)=2.23, p<.05, BF=1.73). Thus, explicit memory about previously encountered haiku is increased when the fragment is positioned in the first line.

Given the relatively small proportion of ‘familiarity’ responses and, associated with this, missing data per participant and poem (haiku type x cut position) condition for this response alternative, it was not viable to carry out a detailed (i.e., poem-category specific) analysis of how memory is linked with eye-movement measures. Separate rm ANOVAs of the first-, second-, and thirdpass dwell times (per word), with the factors *memory response* (recollection vs. familiarity) and *line*, revealed no significant effects, but only some tendencies. For third-pass reading, poems that were correctly recognized (as having been read) with recollective experience, rather than a feeling of familiarity, tended to be associated with longer dwell times in line 1 (223 ms vs. 129 ms), but without a difference in line 2 (151 ms vs. 185 ms) or line 3 (149 ms vs. 130 ms) (interaction memory x line: F(2,20)=3.19, p=.06, BF=1.96). For the second pass, there was some (non-reliable) tendency for explicitly recollected poems (vs. poems recognized as merely familiar) to be associated with increased overall dwell times (per word) (main effect of memory response, recollection vs. familiarity: 209 ms vs. 168 ms; F(1,10)=1.78, p=.21, BF=.58). This pattern tentatively suggests that rereading, and especially re-reading of line 1, plays some role for developing an explicit memory for the read haiku.

Next, we examined the relationship between memory measures and subjective ratings of haiku difficulty (68% of the haiku were rated ‘low’ in difficulty) and, respectively, the extent to which an understanding was achieved (understanding achieved was rated to be ‘low’ for 60% of the haiku). A poem was considered as ‘easy’ (or, respectively, ‘difficult’) to understand if it received a (difficulty) rating of 1 or 2 (or, respectively, 4 or 5) in phase 3 of the experiment. In case a poem was rated as being of difficulty level 3, it was classed as ‘easy’ (or ‘difficult’) if the time required for making the difficulty rating was below (or above) the median of the times across the whole set of the poems. An analogous procedure was adopted for the analysis of haiku understanding achieved. This procedure is justified by the fact that both the ‘difficulty’ and ‘understanding achieved’ ratings were issued faster for ‘easy’ versus ‘difficult’ haiku (two-tailed t test: t(10)=2.87, p<.01, BF=4.04) and for ‘understood’ versus ‘not understood’ haiku (two-tailed t test: t(10)=3.05, p<.01, BF=5.18). [Note 4.]

Of note, there was only a weak correlation between rated haiku difficulty and understanding achieved, likely indicating different underlying variables tapped by these ratings: r=.40, p=.22, lower and upper 95% confidence, CI, limits: -.44, .72; BF=.27. Interestingly, observers’ assessment of haiku difficulty was not systematically correlated with their recognition performance: r=-.04 (p=.92, 95-CI: -.62, .57, BF=.22). By contrast, haiku for which observers achieved an understanding were more likely recognized with ‘recollective experience’, rather than being experienced as just ‘familiar’: r=.57 (p=.06, 95-CI: -.04, .87, BF=1.21). A breakdown of the data, though, showed a significant correlation between haiku understanding and memory performance only for juxtaposition haiku (r=.66, p<.05, 95-CI: .09, .90, BF=2.58), but not context–action haiku (r=.40, p=.22, 95-CI: -.43, .73, BF=.28). Further, the relationship between haiku understanding and recognition performance was modulated by the placement of the cut: the correlation was significant for L.1-cut haiku (r=.65, p<.05, 95-CI: .08, .89, BF=2.34), but not for L.2-cut haiku (r=.48, p=.13, 95-CI: -.16, .83, BF=.69).

In terms of the two subjective ratings (‘haiku difficulty’ and ‘understanding achieved’), juxtaposition haiku were overall rated as being more difficult than context– action haiku (rm ANOVA haiku type x cut position: 2.62 vs. 2.0; F(1,10)=12.70, p<.05, BF=6.51), and L.2-cut haiku were subjectively more difficult than L.1-cut haiku (2.68 vs. 2.00; F(1,10)=12.27, p<.05, BF=40.66), relatively independent of haiku type (juxtaposition: 2.92 vs. 2.32; context–action: 2.44 vs. 1.69; interaction haiku type x cut: F(1,10)=.26, p=.61, BF=.31). Similar effects were found for haiku understanding (rm ANOVA haiku type x cut position: significant main effect of haiku type: F(1,10)=8.98, p<.05, BF=3.76, juxtaposition vs. context

As concerns the relation of the subjective ratings (of ‘haiku difficulty’ and ‘understanding achieved’) to the eye movement patterns, again, because of missing data, for both measures, we examined only the rating (low/high) x line (1, 2, 3) interactions (2 x 3 rm ANOVAs) for the first-, second-, and third-pass reading. For haiku difficulty, the ANOVA of the dwell times (per word) revealed no effects whatsoever. For understanding achieved, the ANOVA revealed a potentially interesting interaction for the second- and third-pass dwell times (2nd pass: F(2,20)=19.86, p<.01, BF=626; 3rd pass: F(>2,20)=3.65, p<.05, BF=1.33): more time (per word) was spent in line 1 of haiku for which an understanding was achieved versus not achieved (2nd pass: 295 ms vs. 160 ms; 3rd pass: 239 ms vs. 146 ms) [Note 5]. Thus, as for explicit, recollective memory, re-entering line 1 for a second or third time would appear to play a role for achieving an understanding of the haiku that is being read.

## Discussion

Next, we summarize the main results of the present study and point out their implications for understanding how eye-movement patterns shape the way the meaning of ELH (and perhaps poetic texts in general) is construed. Finally, we comment on the limitations of the present study and provide an outlook on further work required to develop this line of research further.

### Summary of results and implications

(i) *General effect of cut position: more time spent on fragment line.* The main finding was a *cut effect*. The position of the cut has a major, and general, influence on the eye-movement pattern, that is, on the way readers allocate attention over the poem: statistically, more reading time per word is spent on the fragment line (line 1 in L.1-cut poems and line 3 in L.2-cut poems) than on (each of) the phrase lines, whatever the type of haiku (context– action or juxtaposition) and wherever the cut is placed (at the end of the first or the second line). This pattern is already evident when we look at the first reading of a line (first-pass reading), as well as when the reader re-enters the line for the first or the second time. For instance, in first-pass reading, the total dwell time per word is some 400 ms for the fragment line, as compared to only around 250 ms for the phrase lines. Thus, from the pattern of dwell times, we can deduce where the cut is in the haiku.

Considered along the lines of background and foreground features, perhaps the extended time spent processing the fragment is due to the reader encountering the cut, which acts as a foregrounding, attention-capturing feature. This puts the reader into a more disfluent reading mode, characterized by an increased number of (progressive and regressive) eye movements within, and movements from other (phrase) lines to, the fragment line. The fragment is thus ‘pivotal’ for global meaning construction: the eye, and attention, tends to dwell on and return to the fragment where the ground is laid for the integration of the juxtaposed images.

(ii) *Differential cut effects between L.1-cut and L.2cut haiku*. This general cut effect (see point (i) above) was modulated by the position of the cut: relatively more time per word was spent on the fragment line when the cut was encountered at the end of line 2 compared to when it was encountered at the end of line 1, and this was the case independently of the type of haiku. For instance, in first-pass reading, the total dwell time per word in the fragment line was 470 ms for L.2-cut haiku, but only 330 ms for L.1-cut haiku. This may be taken to indicate that the disorienting, attention-capturing effect of encountering the cut is greater in L.2-cut haiku. Assuming that the phrase lines (1 and 2) of the poem are processed in a relatively fluent, forward-gliding (background) mode (see point (v) below), encountering the fragment in line 3 gives rise to surprise. This, in turn, enforces a foreground mode of processing, attempting to resolve the surprise by extended processing of the fragment (involving an increased number of regressive eye movements) and reconsideration of context, as well as back-tracking to the phrase (lines 1 and 2) and renewed forward-scanning and appraisal of the fragment (line 3). By contrast, when the cut is encountered early on, at the end of line 1, it might immediately draw attention to the grounding context of the poem in the fragment, so that the subsequent phrase lines 2 and 3 are already read in a foreground mode. Further processing, however, also involves a good amount of back-tracking to the fragment (in line 1) and renewed appraisal of the phrase (in lines 2 and 3), though less compared to L.2-cut haiku.

(iii) *Differential eye-movement patterns between context–action and juxtaposition haiku*. While the general cut effect, and its modulation by position, is shared by context–action and juxtaposition haiku, there are also subtle differences between the two haiku types. In particular, the cut effect (extended time spent in the fragment line) is more pronounced for juxtaposition than for context– action haiku, whether the cut follows line 1 or line 2. For instance, in first-pass reading, the average dwell time per word in the fragment line is 360 ms for context–action haiku, but 470 ms for juxtaposition haiku. In other words, the cut effect is modulated by the strength of the (functional-)conceptual distance or discrepancy between the two parts, which is generally greater for juxtaposition than for context–action haiku: the greater the gap between the two images/parts, the more time is spent on working out the meaning implications of the fragment (line).

More insights into the ongoing processes of meaning construction (including their pacing) may be gained by looking at the reading dynamics in the various (first-, second-, and third) passes at the poem and, importantly, by considering the forward- and backward-directed (re-) reading activity together.

(iv) *First-pass reading dynamics*. In the first pass at a haiku, scanning is predominantly forward-directed and focused on the fragment line in both context–action and juxtaposition haiku – as well as on the first phrase line in (both L.1-cut and L.2-cut) context–action haiku, which opens up the action (the majority, 63%, of context–action haiku contained a verb in phrase line 1, which compares with 11% of those haiku with a verb in phrase line 2 and 26% with no verb in either phrase line). Within-line regressions, which are relatively infrequent in lines 1 and 2 (1/4 ratio of regressions to progressions), are concentrated on the third line in all conditions, with re-fixation probability and dwell time approaching pro-fixation probability and dwell time (near 1/1 ratio). This pattern – of relatively fluent (mainly forward-directed) sampling of lines 1 and 2 and more disfluent (more balanced forwardand backward-directed) sampling of line 3 – is perhaps indicative of a first attempt to integrate the haiku’s parts, or form a hypothesis about the haiku’s meaning, at the end of the first pass. In L.2-cut haiku generally, and especially in L.2-cut juxtaposition haiku, the final-line refixations add substantially to the pro-fixations, yielding a very marked ‘dwell’ on the fragment line in the aggregated data (see Table 1 and point (ii) above).

(iv) *Second- and third-pass reading dynamics*. The second- and third-pass reading dynamics are more diverse, permitting a number of condition-specific rereading patterns to be discerned.

For *L.1-cut haiku* (of both the context–action and the juxtaposition type), there is extensive re-sampling of the fragment line (line 1), with more regressive than progressive activity within this line (roughly 3/2 ratio); this is indicative of a disfluent mode of reading, and perhaps of a secondary resolution attempt within this line (after complete first-pass sampling). By comparison, there is only little and/or short re-sampling of the subsequent phrase lines (with a relative balance of regressive and progressive movements within these lines), perhaps to confirm a hypothesis derived from re-reading the fragment line. This pattern essentially recurs in the third pass, though this time with a less marked focus on the fragment line, indicative of a more ‘confirmative’ mode of processing. This is consistent with the idea that when the grounding context (the fragment) is encountered upfront, in line 1, the subsequently encountered phrase (lines 2 and 3) can be re-processed relatively ‘linearly’, in light of the fragment.

While both L.1-cut context–action and L.1-cut juxtaposition haiku share these re-reading dynamics, differential patterns emerge between L.2-cut context–action and L.2-cut juxtaposition haiku.

In *L.2-cut context–action haiku*, second-pass resampling starts with the first phrase line (the action component), with a marked dominance of re- over profixations within this line (roughly 5/2 ratio) – a disfluent reading mode, suggestive of a second resolution attempt (after the dwell on the fragment line at the end of the first pass). This is most likely followed by a progression to the fragment line (i.e., line 3) rather than one to the second phrase line (i.e., line 2), though this time with profixations dominating re-fixations (roughly 5/2 ratio) – indicative of a more forward-directed scanning, perhaps to confirm some already formed resolution hypothesis. Third-pass re-sampling exhibits a similar pattern, though scanning is now predominantly forward- (rather than backward-)directed in both the first phrase line and the fragment line, indicative of a more fluent, perhaps ‘confirmatory’ reading mode. Thus, L.2-cut context–action haiku appear to be resolved by extensive revisits to phrase line 1, as well as work on the fragment in line 3. This would suggest that the reader attempts to work out the impact of the fragment (which provides the grounding context and is encountered at the end of the first pass) on the phrase. This requires that the phrase be re-processed in the light of the fragment, which can bring about a shift in the phrase’s meaning (which is subsequently checked in a re-sampling of the fragment line).

In *L.2-cut juxtaposition haiku*, by contrast, secondpass reading is likely to return to the second (rather than the first) phrase line, with a dominance of pro- over refixations within this line (roughly 2/1 ratio), indicative of relatively fluent, forward-directed scanning. This is likely followed by a progression to the fragment line (line 3), with some dominance of progressive over regressive movements but balanced dwell times, suggesting that a final resolution is attempted in the fragment line. This pattern essentially repeats in the third pass, now with a dominance of forward- over backward-directed activity (roughly 2/1 ratio) in both the second phrase line and the fragment line, again indicative of a more fluent, ‘confirmatory’ mode of reading.Thus, L.2-cut juxtaposition haiku appear to be resolved by readers focusing on the fragment part (i.e., line 3), with comparatively few and/or brief revisits to the phrase part (especially to the second phrase line, i.e., line 2). This would suggest that the meaning of the phrase part has been relatively fixed/worked out in the first pass, and the juxtaposition is resolved mainly by dwelling on the (startling) fragment part.

One (perhaps somewhat puzzling) finding is that the line that receives most re-processing is the first phrase line in L.2-cut context–action haiku, but the second phrase line in L.2-cut juxtaposition haiku. The reason may be that, in L.2-cut context–action haiku (e.g., “**picking stones** / from the lentils ... / winter dusk”; see Figure 1), the first line is more important for the action specification than the second line (2/3 of L.2-cut context–action haiku contained a verb in this line), and/or that the second phrase line is syntactically more integrated with the first one, so that the second line can be taken in relatively fluently once the first line has been processed. By contrast, in L.2-cut juxtaposition haiku (e.g., “photos of her father / **in enemy uniform**— / the taste of almonds”; see Figure 1), the syntax of the phrase lines is often more elliptical or fragmentary, perhaps with the second phrase line providing more information content, as a result of which this line receives more extensive processing in the attempt to link the phrase with the fragment in this type of haiku. – As it stands, this is a post-hoc account that would need to be confirmed with a more extensive sample of haiku to explore differences between noun- versus verb-based constructions of the phrase component.

(v) *Link between haiku understanding and recollective memory.* There were some further, potentially interesting findings concerning a link between memory for the read haiku, as assessed in the post-reading memory test (i.e., how well, in terms of recollective experience, the haiku was recognized as previously read), and haiku understanding achieved, as assessed in the final subjective ratings.

Overall, with a recognition success of 86%, memory for read haiku was quite high, and successful recognition was largely associated with (self-stated) explicit, ‘recollective’ experience rather than just a vague feeling of familiarity. There were no statistically robust effects linking memory with eye-movement measures, that is, from the eye-movement pattern alone, one cannot tell whether a given haiku was later explicitly recognized (i.e., recollected) as read, or just judged as (vaguely) familiar. Interestingly, participants’ assessment of haiku difficulty was wholly uncorrelated with their recognition performance. But haiku for which participants achieved a better (self-rated) understanding were more likely recognized with recollective experience, rather than being experienced as just familiar [Note 6].

Concerning links of both ‘memory’ and ‘achieved understanding’ measures with eye-movement behavior, the only discernible trend was that poems that were recognized with recollective experience (as compared to a feeling of familiarity) and poems of which an understanding was achieved were associated with longer dwell times in line 1 on (second- and third-pass) re-reading. Potentially of interest in this context is that, at the end of reading, the eye often re-entered line 1, contributing to the dwell time in this line. While this return saccade (to the beginning of line 1) may have been made in anticipation of the fixation point for the next poem, it may also serve a ‘meaning wrap-up’ function (cf. 12 ), which may be important both for finalizing an understanding of the haiku and for memory formation.Note that Carpenter and Just ( 12 ) attributed the extended gaze durations they observed towards the end of the reading of (ordinary) sentences to meaning wrap-up. With our text material, and given the way successive poems were presented on the monitor, it is conceivable that such fixations may occur after return to the beginning of the poem. This is, of course, speculative and would need to be corroborated in future work.

Note that recognition memory for read texts can be based on different kinds of (memory) representations (e.g., 103 ): semantic representations/situation models (enriched by background knowledge) that result from comprehending the poem, or more surface-level/formrelated representations. Indeed, as regards recognition memory for (longer forms of) poetry,Yaron ( 111 ) proposed that with ‘difficult’/‘obscure’ poems, readers will rely strongly on surface-level representations (i.e., exact representations/‘copies’ of the linguistic form of the read texts), presumably because they are unable to construct complete/coherent (higher-level) semantic representations/situation models for these poems. With ‘easier’ poems, by contrast, readers will rely more on the higherlevel, less form-based representations during memory testing. (This would also explain why Yaron 111 found better, i.e., more precise literal recall performance for ‘difficult’ than for ‘easy’ poems: the semantic/situation model representations readers consult with easy poems do not contain information of the exact wording of the poems!) Based on our material (short poems) and data, we cannot tell exactly what level of representation(s) our readers relied on for making their memory response: did they rely on higher-level representations for poems that they indicated they had understood, and on surface-level representations for poems that they felt they had not understood?

Nevertheless, the fact that ‘understanding achieved’ was predictive of memory performance (and not poem ‘difficulty’ as such) makes it likely that processes of actually forming a semantic interpretation, and the images/feelings/thoughts associated with this, contributed to poem recognition with recollective experience. This would run counter to the standard *levels-of-processing* notion ( 18 ), according to which the depth level adopted during the processing (i.e., here, the meaning-oriented mind set with which a poem is approached) is the prime, if not sole, factor determining memory performance. However, it would be consistent with other views according to which recollective experience may be associated with experiencing an ‘aha’ moment (i.e., actually resolving the haiku’s meaning, rather than just striving to resolve it) and the (feelings of) reward associated with this – where the ‘aha’ experience and reward may engender a *self-reference effect*(e.g., 96 ) and promote episodic (i.e., *autonoetic*) memory formation and retrieval (e.g. 100, 109 ). Reward may be necessary for, or at least reinforcing, memory consolidation and thus (explicit) retrievability, via activation of the mesolimbic dopaminergic (MLDA) system (e.g., 79 ). Lack of resolution, on the other hand, may also be associated with motivated suppression of memories associated with failure. Again, of course, these are hypotheses that would need to be examined in future work (possibly using recall tests which would be more diagnostic as to the representations on which memory performance is based).

(vi) Taken together, these findings – in particular, the effect of cut position – are quite robust and cannot be reduced to other factors that were not explicitly controlled in the experiment (such as word length and other lexical and supralexical factors). In particular, the observed patterns of re-fixations cannot simply be explained based on general linguistic principles: close scrutiny of individual readers’ scan paths revealed no evidence of any strong, systematic, and inter-individually coherent effects of particular linguistic features – like phoric elements (e.g., pronouns, definite determiners, etc.) in general, and phoric elements without (explicit) in-text antecedents in particular (e.g., *he* and *him* in Melissa Allen 1 , see Figure 1) – which, in the literature (e.g., 111, 76 ) have been identified as potential triggers of regressive saccades in reading. Although those linguistic features did trigger regressive saccades in many instances in our data, a lot of other words and features did so as well. In addition, regressions were not necessarily directed to phrase-initial elements followed by intra-line profixations on the phrase; rather, in many instances, the saccade following a regression was actually directed leftwards (i.e., counter the reading direction). Finally, most of the typical ‘suspects’ triggering increased regressive and (in turn) progressive saccades are relatively evenly distributed across the haiku conditions used in the present study, thus making it unlikely that the haiku-type and cut-position effects that we found are systematically confounded by such factors.

(vii) There are a number of further, general observations (in part also deriving from inspection of individual scan paths) worthy of note. Overall, the reading patterns are markedly non-linear: the numbers of pro- and regressions – within and, in particular, across lines – appear higher with haiku than with most other texts (e.g., about one third of cross-line regressions as compared to the usual 10–15% reported by Rayner ( 83 , see above)). In addition, there was a very marked tendency to skip function words. While this phenomenon has been reported to occur with up to 50% of function words in ‘standard’ texts (which has been reported to occur with up to 50% of function words in standard texts; (94), on many trials in our study, readers started by jumping from content word to content word (i.e., they skipped almost all of the function words) and only took in the text as a whole on later reading(s) of the same poem. Further, in addition to ‘regress-and-progress’ sequences of re-reading eye movements, there were also many instances of ‘regress-andregress’ movements, that is, sequences of movements starting from line 3, with one subsequent fixation in line 2 and the next one in line 1. Overall, this spatially distributed reading pattern might be characteristic of reading haiku (or perhaps of short poetry in general; see also 61, 88, 111, 112 ). And the focus on content words might, to a certain extent, be the result of the partly fragmentary or elliptical syntax in the haiku, as well as of haiku being a “poetry of nouns” (see, e.g., 49 ).

(viii) The main effect of the position of the cut (adding several hundred milliseconds of dwell time per word to the fragment line), as well as the main effect of haiku type are of particular interest, because they permit us to tell from the eye-movement pattern alone which haiku (in terms of type and cut position) is being read. The haiku poet might consider the effects of cut position and haiku type as ‘a given’, as the strength of the juxtaposition and the positioning of the cut (i.e., foregrounding techniques) were techniques designed to induce in the reader this particular pattern of non-automatic processing and meaning resolution. In the cognitive-poetics literature, however, this result has novelty value. While some stylistic and form features typical of poetic texts, like the spatial layout of the text on the page ( 88 ) or the stylistic device of enjambement ( 61 , ; see also 10 ) have been identified to have specific effects on eye movements during reading, there have not been other findings of signature eyemovement patterns reflecting the more content-related features of an unexpected sharp thematic or imagistic *turn* in poetry, as is, for instance, also characteristic of sonnets ( 8 , p. 10). While such *turn* or *volta* effects might still be found in other poetry in future research, the fact that we were able to establish such a signature pattern in the present study (even though we used readers that were naïve with regard to the genre of haiku) suggests that haiku – of the particular sort and quality found in leading ELH journals, which we used in the present study – are a particularly potent material for studying processes of literary meaning construction in neuro-/cognitive poetics.

### Limitations and outlook

In what follows, we discuss (some of) the limitations of the current study and provide pointers for future research.

Implications for NCPM. The present findings have implications for the neuro-cognitive poetics model (NCPM; e.g., 45 ). Using an eye-movement measure deriving from NCPM, we could show that ‘reading fluency’ – assessed in terms of the ratio of forward- to backward-directed oculomotor activity within lines – exhibits patterns characteristic of particular classes of haiku, including systematic changes in the speed and, thus, pacing in which particular lines are scanned for the first time and then (selectively) re-sampled – to construct and check global meaning. In this sense, the NCPM provided us with both a framework to explore the reading of haiku and ‘tools’ that permitted us to depict some (across haiku types and cut positions) relatively stable patterns of the reading dynamics, in terms of shifts between (predominantly) ‘background (BG)’ /fluent and (predominantly) ‘foreground’ (FG)’/disfluent processing modes – central concepts within the NCPM. Given this, our findings can be taken to reinforce some of the basic distinctions fundamental to the NCPM. Arguably, though, to formulate more precise, ‘local’ hypotheses as to oculomotor activity, one would have to ‘zoom in’ at the level of individual poems and poem lines, rather than more ‘global’ classes of poems and structural characteristics of such classes – which was the level at which the present study was pitched. This remains a task for future research.

As for the present findings: given that ELH is still a relatively uncommon literary genre (despite its rapidly increasing popularity), the responses to haiku type and structure that we observed in ‘naïve’ readers cannot be attributed to overlearned ways of approaching this kind of poetry. This marks our research as different from many other studies that build on conventional/highly familiar types of text. Taking this into account, it would appear non-trivial that our findings are interpretable in line with FG-BG theory, which is at the core of the NCPM: they may be taken to suggest that this reader behavior characterizes ‘literary reading/processing’ in general, that is, even if participants have no awareness of genre-specific formal features and/or of text-type-associated (pragmatic) knowledge structures acquired from previous (reading) experiences. (See also section Developing a ‘sense’ for haiku below).

Role of linguistic features.While most of the effects we found in terms of cut position and haiku type are quite robust and not readily reducible to other (specific) linguistic features of the texts (see above), interpretation of the more subtle, higher-order (haiku type x cut position x line) interactions may be limited by our restricted sample of (48) haiku presented for reading (as well as the small number of participants). In particular, we cannot rule out that subtle linguistic differences between L.2-cut context–action and L.2-cut juxtaposition haiku (such as increased syntactic integration of the phrase lines in the former as compared to the latter) played some role for producing these interactions. This would need to be examined in future studies. Note though that, based on the present data, we can already conclude that the amount of variance explained by other linguistic factors is minor compared to that generic to the form, such as the very prominent cut-position effect. Nevertheless, a systematic analysis of (micro-)reading patterns – at the level of individual poems, and with a representative sample of poems – would be an interesting task for future research.

Examining what is ‘unsaid’ in poetry. Beyond the linguistic surface level, haiku, by their very nature, are brief, highly condensed poems that necessarily leave many things unsaid: “... the true subject of a haiku is never mentioned in the haiku. It is what a haiku implies that makes it a great or worthless haiku” (R. H. Blyth, quoted in Kacian, 49 , p. 39). Much of haiku’s impact derives from the ‘gap’ between the juxtaposed images, which the reader has to fill in to achieve closure of the meaning Gestalt – a process that, according to haiku theorists, is driven by the energy contained in the images themselves [Note 7]. Like in vision, where the perceptual Gestalt formed includes elements not actually present in the distal stimulus, the unsaid is ‘elaborated’ in the meaning Gestalt constructed from the images presented. This raises an interesting question (for neuro-/cognitive poetics), namely: “to what extent is eye tracking a suitable method for gauging what is not seen, or absent, on a page?” (Jacobs, personal communication, December 2016). As for the present study, of course, our eye-movement analyses – examining oculomotor activity collapsed across haiku, or haiku classes, and readers – cannot tell how a specific reader filled in the gap in a specific poem, but we gleaned information about some of the major sampling strategies (of intial reading and selective re-rereading) that readers adopt for arriving at an ultimate solution. In this sense, our analyses go beyond the reading of the words that make up a poem. However, to paraphrase Jacobs ( 45 ), this may not be far enough: “neurocognitive poetics research needs testable hypotheses about what those things ‘absent’ from a text elicit in a reader’s mindbrain” (p. 6). Accordingly, appropriate analyses would need to be conducted at the level of individual readers, taking into account their “‘apperceptive mass’ ( 48 ), i.e., their knowledge (e.g., semantic and autobiographical memory), motivations, expectations, preferences” ( 45 , p. 6) ; and at the level of individual poems, taking into account their larger meaning potential (i.e., multiple meanings). On the reader side, such analyses would conceivably involve direct poem-specific analyses of understanding achieved or (reproduction) memory of the meaning constructed (cf. 111 ). And modeling of how readers (creatively) arrive at something new from initially (seemingly) incompatible information might profitably draw on established (cognitive-linguistics) theories, notably Conceptual Integration/Blending Theory (e.g., 26, 27, 28, 101 ). Arguably, though, haiku might provide an apt material for such analyses and modeling attempts in future work.

Specifically of interest in this context would be to investigate more closely the reading of haiku as a function of the distance (the ‘gap’) between the images in the phrase and fragment parts in both (context–action and juxtaposition) types of haiku. One way to approach this is to have a representative set of haiku (independently) rated in terms of the magnitude of this distance – or alternatively, derive a measure of (content) word predictability as an indicator for the ‘surprise value’ or ‘strength’ of the cut (e.g., for L.2-cut haiku: the less predictable the first (content) word in the fragment is from the last (content) word in the phrase, the stronger the cut) – and then examine the reading eye-movement pattern as a function of these measures.

Three-line versus one-line ELH. Given the very pronounced cut-position effect observed in the present study, it would be interesting to compare, in future work, the reading of normative, three-line haiku (examined exclusively in the present study) with that of one-line haiku (*monoku*). In three-line haiku, the cut position is typically clearly indicated, and maybe additionally emphasized by an explicit cut marker (in fact, it would be interesting to explore the effects of different types of cut in three-line haiku – with or without punctuation, and the type of punctuation – on processes of meaning construction). In monoku, by contrast, the position of the cut is often ambiguous, with this ambiguity being a design feature: it is deliberately introduced by the poet, overloading the poem with multiple ambiguities; the best monoku characteristic of the form is designed to permit, and induce, play with different segmentations of the poem’s elements and thus different (re-)constructions of the haiku’s meaning. An additional technique of interest in monoku is the omission of the fragment from the poem: rather than juxtaposing two images in a tense relationship, in monoku “a single image is extended or elaborated into a second context, often implied” ( 50, 51 ) – a technique which complicates the reader’s task of meaning analysis and construction and renders monoku a particularly valuable comparative form to the normative haiku composed of fragment and phrase. This may also have a bearing on memory for the haiku read: retention may be impeded for monoku (compared to three-line haiku) because of a greater difficulty to achieve closure; or, because these poems ask for more, they may engender improved retention. Thus, examining how this potential for multiple meanings is reflected in the reading eye movements, as well as in memory measures, may be best assessed using one-line haiku.

Developing a ‘sense’ for haiku. The participants in the present study were naïve readers, who, to start with, had little ‘sense’ for haiku: they were all new to this genre of poetry and had to learn during the experiment how to read and achieve an understanding of ELH. Experienced readers may have acquired, and thus have at their disposal, special strategies of resolving, say, haiku with a cut after line 2 or haiku with a greater (conceptual) distance between the images juxtaposed in the phrase and fragment parts (i.e., haiku with which our naïve readers tended to struggle, as evidenced by their ratings of haiku difficulty and understanding achieved). In this regard, the fact that we presented the various (haiku type x cut position) categories of poems in random, intermixed order potentially limited the ability of our naïve readers to learn how to ‘read’ such haiku: learning may be hampered when one has to permanently switch between poem (haiku type x cut position) categories (see e.g., 10 , for the development of sub-genre specific reading strategies). This issue would need to be addressed in future, purposedesigned research, for instance, by blocking haiku type and cut position (as compared to presenting them in random sequences). In this context, it would also be of interest to compare naïve readers of haiku with a group of experts who have a working knowledge of the poetic techniques and devices employed by the poet. In fact, from a developmental point of view, learning to read (and write) haiku might be particularly educational for developing ‘the poetic sense’ in general (in line with Jacobs & Kinder’s 45 suggestion of the useful role that micropoetry might serve in this regard).

Aesthetic trajectory and aesthetic liking.Aesthetic appreciation – one of the key issues in poetry reception (including the NCPM; 45 ) as well as haiku theory (see, e.g., 45, 54 [Note 8]) – was not directly explored in the present study. Given our (secondary) focus on memory performance as an indicator of the depth of processing and construction of meaning (‘understanding achieved’), we had decided to limit our study to these more ‘cognitive’ factors rather than including further subjective ratings of aesthetic appreciation and the feelings associated with it (which is, in itself, a complex issue; see, e.g., 31, 46 ). In contrast to (direct) oculomotor measures, (indirect) measures such as changes in pupil diameter might be more readily related to aesthetic liking and memory processes, at least under certain conditions (e.g., 63, 105, 64 ). However, since we only collected (subjective) memory responses, but no ratings of aesthetic experience(s), we would be able to relate pupillometric measures only to the former, but not to the latter, and so be unable to discern influences of the two on changes of pupil diameter. Reasonably, however, one might assume that (our measure of) ‘understanding, or closure, achieved’ (rather than perceived ‘haiku difficulty’) might well correlate with aesthetic appreciation – implying that aesthetic experience correlates with, or contributes to, explicit (‘recollective’) recognition of the respective haiku. These are issues that await further, dedicated research [Note 9]. Note though that this research might profitably be guided by our findings of the differential ‘pacing’ with which haiku are read in the various passes. We predict that pupillometric changes would more be informative about both memory performance and aesthetic experience during the second or third reading passes, during which, based on the present data, much of the work is done to construct global meaning.

‘Musicality’ in haiku and aesthetic liking. While haiku tend not to use rhyme (because “rhyme remains such a compelling device that its presence in this fragile form is often overpowering”; 49 , p. 84), elements of musicality, specifically stress and rhythm as well as sound and timbre, are carefully crafted by the poet to ‘energize’ the images in a haiku and create the ‘spark’ between them. To quote Kacian ( 49 ): “... often we are attempting to give voice to the wordless, and it is only through mastery of the musical [i.e., rhythm and timbre] elements of a poem that we can approximate the effect of the experience upon us” (p. 89). In ELH, “it is unusual to have fewer than one or more than three stresses per line”, with “stresses [occupying] the center of attention in each line, and … the unstressed syllables [serving] to bridge the time between these stressed moments, creating a rhythm specific to the poem” (p. 87). “… in such a brief [form], what matters … is that the rhythm be suggestive of the experience, that it contain the energy of the moment and attract the reader to it” (p. 87). As to tonal quality, “some syllables are susurrant, some percussive, some nasal. [Their] combination … across the duration of the haiku account[s] for its timbre. ... In each case, we are choosing words not just for meaning, but for tonal quality” (p. 87). This combination of rhythm and timbre elements is what makes the sound of a haiku. Given the complexities involved, again, future, dedicated research would be required to examine the effect of these musical elements (including aspects of ‘phonological iconicity’ and ‘mental sound’), on haiku reception and aesthetic liking (for effects of these elements even in silent poetry reading, see 2, 14, 74 ).

Neuro-cognition of haiku reading. Another limitation is that the present study relied solely on eye-movement, coupled with recognition memory, measures to examine the reading of haiku. While such measures are highly informative of key mental processes going on while reading haiku, they would need to be augmented by ‘brain’ measures to acquire a more complete, and complementary, ‘neuro-cognitive’ picture of the reading process. Particularly pertinent would be measures of semantic incongruity detection (like the N400 component of the EEG; for review, see 66) and/or measures of surprise resolution (like EEG components and brain-oscillatory activity profiles associated with the ‘aha’ moment; for review, see 62 ). The difficulty, though, is that EEG components are more difficult to extract in dynamic reading situations that involve eye movements (which induce ‘artefactual’ electrical signals at the scalp surface). However, the methodological challenges associated with this are being overcome (e.g., 21 ). Interestingly in this regard, the German poet Durs Grünbein ( 38 ) called for a poetry full of images (metaphors) rich in “factor N400”, that is, rich in foreground features (FG) that evoke “neurolinguistic clashes” and act as “brainphysiological attention catchers”. Future studies may show that this is particularly true for haiku.

Haiku as paradigmatic study material. Finally, by suggesting ‘haiku as paradigmatic study material’ (see Introduction), we do not wish to imply that the study of haiku reception should replace studies of longer literary/poetic texts – not least for reasons of ecological validity, as the genre of haiku occupies but a small niche within the realm of literary/poetic forms (though see our remarks on potential advantage of studying the reader response to such a relatively unfamiliar genre of poetry in section "Implications for NCPM" above). Arguably, however, the study of short poems, like haiku, allows for more systematic variation of experimental conditions (in our study: haiku types and cut positions) than would be possible with longer texts – and in this sense, it provides an interesting paradigm for exploration in future studies. With longer texts, of course, innovative quantitative narrative analysis (QNA) tools (e.g., 31, 46 ) provide an apt basis for relating ‘processing’ (reflected in eyemovement measures, pupillometric and peripheralphysiological measures, BOLD measures, etc.) to the variables measured by these tools. However, from an experimental point of view, systematic variation of certain variables and observing the effects of these variables can provide additional information beyond the ‘relationships’ revealed by alternative approaches. Ultimately, though, it would be ideal to combine both approaches – such as applying QNA to haiku – in future research (see also 110 ).

Last but not least: Given their typical content (images from everyday contexts), haiku might also be particularly well-suited for testing the NCPM’s ‘*mood empathy hypothesis*’, that “poems expressing moods of persons, atmospheres, situations or objects should engage readers to mentally simulate and affectively resonate with the depicted state of affairs, thus facilitating immersive experiences” ( 46 , pp. 91-92). This, too, awaits further research.

### Conclusions

The present study aspires to open up a new terrain for neuro-/cognitive poetics: English-language haiku as a paradigmatic material for studying meaning (re)construction in the mind-brain. The results demonstrate that, out of the elements created by the poet (fragment, phrase) and skillfully placed into a dynamic relationship using such techniques such as the juxtaposition of images or cut, the reader is made to (re-)create in her/his mind one pattern (or several, alternative patterns) from the within the poem’s wider meaning potential. This interactive process between the poem and the reader, which may culminate in an ‘aha’ experience in the reader, gives rise to a characteristic pattern of eye movements and fixations across the text, indicative of the type of haiku (context– action vs. juxtaposition) and the position of the cut (after line 1 vs. after line 2). Moreover, in a memory test administered after reading, readers reported a more explicit (i.e., conscious) experience of having read a particular haiku if they had been able to understand the poem. This suggests that an ‘aha’ experience may enhance memory consolidation and later retrieval. Further work, going beyond eye-movement and memory measures, is necessary to examine how these processes arise in the reader’s brain.

## Notes

Note 1. Relating the oculomotor measures to ‘words’ is in line with the consensus in the eyemovement literature ( 25, 83 ) that the word, bounded by spaces, is a basic perceptual encoding unit in the reading of alphabetic scripts. Admittedly, though, the concept of ‘word’ as such is far from unproblematic (see, e.g., 90 ): (linguistic) units of representation and, potentially, also of processing might be larger (or smaller) than words; for instance, they might take the form of phrase- or sentence-level constructions or chunks, and might even differ in size and nature (e.g., degree of schematicity) between individuals and/or between instances of use/processing (see e.g., 39 , p. 125 and p. 142), as is suggested by principles and insights central to usage-based cognitive linguistic and construction grammar frameworks (e.g., 37, 9, 69, 19 ). Arguably, though, such suggestions are still to be subjected to systematic theoretical modeling and, in particular, empirical investigation – justifying our pragmatic approach (for the purposes of this study) of relating eye movements to the number of words per line.

Note 2. Counting a cross-line regression to a particular line as a regressive movement for this line may appear as somewhat arbitrary (also given that, by definition, cross-line regressions could not occur for line 3). Arguably, however, a regression from, say, line 2 to the beginning of line 1 is similar to a regression from the end of line 1 to the beginning of line 1 (e.g., in both cases, the eye has to assess where it landed, whether it landed correctly, etc.). Also, in fact, there may be different (sub-)classes of intra-line regressions (e.g., is a regression from the end to the beginning of a line similar to a regression from the end of a word to its beginning?). In the present analysis, such fine details go under in the ‘noise’. If we nevertheless find consistent patterns, we can deduce that these are informative.

Note 3. This 2 [haiku] x 2 [cut position] x 3 [line] rm ANOVA also revealed a significant main effect of cut, F(1,10)=10.89, p<.01, BF=1.59 (dwell times were longer for L.2- relative to L.1-cut haiku: 694 vs. 615 ms) and a significant cut x line interaction, F(2,20)=10.54, p<.01, BF=26.04 (for L.1-cut haiku, dwell times were longer in line 1 relative to lines 2 and 3: 718 vs. 587 and 540 ms; for L.2-cut haiku, they were longer in line 3 relative to lines 1 and 2: 875 vs. 582 and 626 ms). Note that, henceforth, we limit the presentation of ANOVA results to the ‘highest’ effects, that is, we do not report main effects or lower-order interactions in case they were qualified by significant higher-order interactions.

Note 4. The reason for collapsing the 5-point rating scales into binary scales was that, averaged across the two (‘difficulty’ and ‘understanding achieved’) ratings, most haiku received ratings of “1” (42.2%), whereas ratings of “2”, “3”, “4”, and “5” were relatively, and increasingly, rare (21.5%,14.3%, 13.1%, and 8.9%, respectively). The latter means that, for instance, there would have been only some 4–5 (‘unrepresentative’) poems that were assigned a rating of “5” – which would have rendered a more fine-grained analysis questionable. Thus, especially also with regard to linking the rating to eye-movement data, we decided to convert the 5-point scales into dichotomous scales.

Note 5. Conversely, spending more time in line 3 of a haiku was associated with failure to understand, rather than successful understanding (2nd pass: 198 ms vs. 275 ms; 3rd pass: 147 ms vs. 165 ms, nonsignificant numerical difference).

Note 6. The latter is qualified by the fact that, in a more detailed analysis, the correlation turned out significant only for juxtaposition haiku (not context–action haiku) and, respectively, for L.1-cut haiku (not L.2-cut haiku). For both juxtaposition haiku and L.2-cut haiku there was also a greater range of variation in participants’ ratings of understanding achieved, which might explain the finding of a significant correlation with memory.

Note 7. To quote Kacian ( 49 ): “We might consider the images to be the two poles of an electrical element, like a Tesla coil, and the relationship between them to be the spark which shoots the gap. The more powerful, clear and certain the choice of images, the brighter and surer the spark ... Our goal in haiku is to find the correct images to serve as poles, and to allow the energy in the things themselves, the images and the language, to provide the spark inherent in them” (p. 56). To power the gap, the skilled poet can draw on a range of techniques – including comparison, contrast, association, verb/noun exchange, sense switching, sabi (understated loneliness mixed with sadness), wabi (values of simplicity), amongst others (see, e.g., 87 ) – that, although ‘invisible to the eye’,bring the images together.

Note 8. As we discussed elsewhere ( 81 ): “In the haiku literature, … ideas of background and foreground abound, often traced to the original Japanese roots of the form, where the poem was presented as an object on an aesthetically enhancing background. It was ‘written’ in ideogrammatic characters, each loaded with references, cultural associations, and layers of meaning. As such, it was primarily viewed rather than read, giving rise to a different mode of experience ( 54 ). While Western English language haiku is predominantly read, several of its elements ‘reach beyond the bounds of what is normally considered language’s terrain into the realm of pictures and even beyond that: unwritten, non-textual and even at times invisible elements contribute to the haiku’s power’ ([ 54 ] p. 51) ... invit[ing] a viewing mode/add[ing] aesthetic value to the poem ( 51 ).”

Note 9. We thank both reviewers for this valuable suggestion for future research.

## Acknowledgments

We wish to thank Melissa Allen, Mark Brager, Sonam Chhoki, and Sandra Simpson for permitting us to reprint their poems in this paper, and Mariam Kostandyan (student assistant) for help with running the experiment. Special thanks are also owing to the reviewers of this article, Arthur M. Jacobs and one anonymous reviewer, for their generous and most insightful comments on an earlier version of the manuscript. Finally, we acknowledge the indirect support of the German Research Foundation (Deutsche Forschungsgemeinschaft). Some of the ideas presented in this report have been included in an article, by Pierides et al. ( 42 ), addressing the community of haiku poets.

## Supplementary Tables

**Supplementary Table S1.** Likelihood with which a line is fixated in second- and third-pass reading (upper and lower half of the table, respectively). FLSD: Fisher Least Square Difference.

**2nd pass**	**L.1 c-a**	**L.2 c-a**	**L.1 juxta**	**L.2 juxta**
**Line 1**	0.59	0.64	0.66	0.59
**Line 2**	0.78	0.73	0.74	0.65
**Line 3**	0.62	0.59	0.57	0.62

**3nd pass**	**L.1 c-a**	**L.2 c-a**	**L.1 juxta**	**L.2 juxta**
**Line 1**	0.32	0.36	0.39	0.39
**Line 2**	0.55	0.55	0.51	0.44
**Line 3**	0.31	0.41	0.30	0.36

FLSD 2nd pass and 3nd pass: .10

**Supplementary Table S2.** Average rank in which a given line was entered in second- and third-pass reading (upper and lower half of the table, respectively). FLSD: Fischer Least Square Difference.

**2nd pass**	**L.1 c-a**	**L.2 c-a**	**L.1 juxta**	**L.2 juxta**
**Line 1**	1.56	1.59	1.48	1.44
**Line 2**	1.38	1.50	1.48	1.56
**Line 3**	2.35	2.45	2.45	2.59

**3nd pass**	**L.1 c-a**	**L.2 c-a**	**L.1 juxta**	**L.2 juxta**
**Line 1**	1.47	1.81	1.52	1.18
**Line 2**	1.38	1.64	1.31	1.61
**Line 3**	1.94	2.02	2.16	1.95

FLSD 2nd pass: .33, FLSD 3nd pass: .22
